# Reduction of alternative electron acceptors drives biofilm formation in *Shewanella algae*

**DOI:** 10.1038/s41522-020-00177-1

**Published:** 2021-01-27

**Authors:** Alberto J. Martín-Rodríguez, José A. Reyes-Darias, David Martín-Mora, José M. González, Tino Krell, Ute Römling

**Affiliations:** 1 Department of Microbiology, Tumor and Cell Biology, Karolinska Institutet, Solna, Sweden; 2 Department of Environmental Protection, Estación Experimental del Zaidín, Spanish National Research Council (CSIC), Granada, Spain; 3 Department of Microbiology, University of La Laguna, La Laguna, Spain

**Keywords:** Biofilms, Environmental microbiology

## Abstract

*Shewanella* spp. possess a broad respiratory versatility, which contributes to the occupation of hypoxic and anoxic environmental or host-associated niches. Here, we observe a strain-specific induction of biofilm formation in response to supplementation with the anaerobic electron acceptors dimethyl sulfoxide (DMSO) and nitrate in a panel of *Shewanella algae* isolates. The respiration-driven biofilm response is not observed in DMSO and nitrate reductase deletion mutants of the type strain *S. algae* CECT 5071, and can be restored upon complementation with the corresponding reductase operon(s) but not by an operon containing a catalytically inactive nitrate reductase. The distinct transcriptional changes, proportional to the effect of these compounds on biofilm formation, include cyclic di-GMP (c-di-GMP) turnover genes. In support, ectopic expression of the c-di-GMP phosphodiesterase YhjH of *Salmonella* Typhimurium but not its catalytically inactive variant decreased biofilm formation. The respiration-dependent biofilm response of *S. algae* may permit differential colonization of environmental or host niches.

## Introduction

A hallmark of members of the genus *Shewanella* is their remarkable respiratory versatility since they can respire on an array of organic and inorganic compounds comprising virtually any electron acceptor more electronegative than sulfate^1,2^. The respiratory capacity of *Shewanella* is reinforced by mediator-directed electron transfer mechanisms enabling the efficient reduction of insoluble substrates such as metal oxides^3^. This respiratory flexibility results in an extraordinary physiological versatility that contributes to the environmental abundance of the chemoorganotroph shewanellae as versatile colonizers of oxic, hypoxic, and anoxic marine and freshwater habitats^2^. Some species, primarily *Shewanella algae* and close relatives such as *Shewanella chilikensis*, have been associated with human disease^4,5^.

Biofilm formation promotes the colonization of biotic and abiotic substrata in *Shewanella* spp. and many other bacterial species^6^. This predominantly sedentary lifestyle is a developmental process initiated by an attachment of single cells that subsequently form microcolonies prior to the establishment of mature biofilms. The intracellular second messenger cyclic diguanylate monophosphate (c-di-GMP) is known to play a key role in the lifestyle switch from planktonic to sessile and vice versa in a plethora of Gram-negative species^7,8^. Integration of extra- and intracellular stimuli modulates different features of this multicellular microbial lifestyle in response to changes in nutrient availability, temperature, oxygen tension, quorum sensing, and various chemical signals^9,10^. For example, reduced oxygen tension differentially regulates biofilm formation in *Shewanella oneidensis* MR-1 and *Shewanella putrefaciens* CN32^11,12^. For *Shewanella* spp., life within a biofilm provides structural and functional advantages, spanning from maintenance of redox balance to enhanced extracellular electron transfer^13–15^. *Shewanella* spp. thrive in hypoxic and anoxic environments including sediments, hypoxic water bodies, and the intestinal tract of fish and invertebrates^16–18^, where these non-fermentative species use some of the most abundant inorganic and organic alternative electron acceptors (AEAs). While biofilm formation on insoluble metal oxides has been well documented^19^, knowledge on the behavioral and physiological biofilm responses upon use of the broad array of AEAs is limited in *Shewanella* species, as is the repertoire of terminal reductases enabling the respiration of AEAs.

Here we show that supplementation of a seawater-mimicking medium with organic or inorganic AEAs induces differential, strain-specific biofilm formation in *S. algae*. Assessment of the respiratory capacity of the strains combined with mutant analyses of the type strain *S. algae* CECT 5071 demonstrated that respiration is required, but not sufficient per se to increase biofilm production upon AEA supplementation. Transcriptome analyses showed that the number of differentially regulated genes positively correlated with the amount of biofilm formed by *S. algae* CECT 5071 upon addition of the electron acceptors DMSO and nitrate. Several of the differentially regulated genes encoded proteins that participate in c-di-GMP turnover. Altogether, our study suggests that differential biofilm formation in *S. algae* strains is governed beyond respiration by the induction of distinct biofilm developmental programs.

## Results

### Alternative electron acceptors promote strain-specific biofilm formation in *S. algae*

**Fig. 1:**
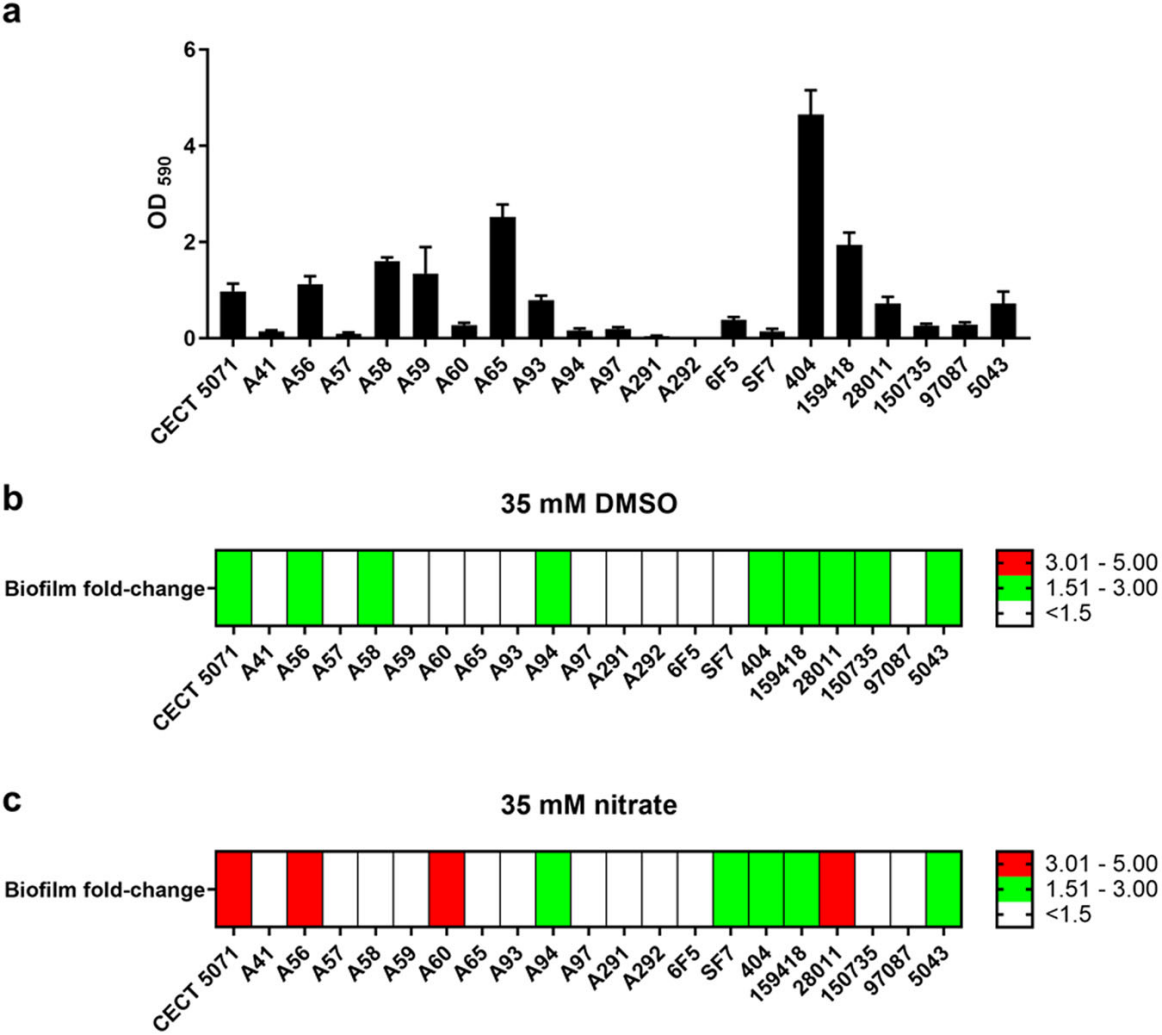
Biofilm formation response of *S. algae* strains in the absence or presence of supplemented alternative electron acceptors. **a** Biofilm formation for 21 *S. algae* strains in MB medium in the absence of added electron acceptors. **b** Heatmap representing the fold-change in biofilm formation with respect to the corresponding non-supplemented control upon addition of 35 mM DMSO. **c** Heatmap representing the fold-change in biofilm formation with respect to the corresponding non-supplemented control upon addition of 35 mM sodium nitrate. Data represent the average of two biological replicates with six technical replicates each.

While screening chemical libraries for biofilm inhibitors, we noticed that an addition of 35 mM DMSO enhanced biofilm formation of *S. algae* CECT 5071 static cultures ~2-fold compared to the non-supplemented control. Since a similar phenomenon had not been observed with other DMSO-respiring bacteria, we reasoned that this phenotype could be specific to *S. algae*. We therefore analyzed DMSO-mediated biofilm formation in a selection of 20 *S. algae* strains from various environmental and clinical sources (Supplementary Table 1). Biofilm formation in plain Marine Broth (MB) varied substantially between isolates, from poor biofilm formers to proficient biofilm producers (Fig. 1a and Supplementary Fig. 1). Biofilm formation values in MB were taken as baseline levels for comparisons upon AEA supplementation. Thus, for 9 of the 20 strains we observed a more than 1.5-fold induction of biofilm formation in response to DMSO (Fig. 1b and Supplementary Fig. 2).

Since the respiratory repertoire of *Shewanella* spp. is broad, we wondered whether other AEAs provoked a similar strain-specific biofilm response. To that end, we analyzed the biofilm response of the type strain *S. algae* CECT 5071 and the other 20 *S. algae* isolates towards 35 mM nitrate, another representative AEA during anaerobic respiration (Fig. 1c and Supplementary Fig. 3). With an increase of more than 4-fold, *S. algae* CECT 5071 showed the most pronounced increase in biofilm formation as determined by CV staining. Further 9 strains, 2 of which are different to those reported above for DMSO, responded with increased biofilm formation to nitrate supplementation.

To ensure that the observed biofilm induction was not due to pH changes upon utilization of the different AEAs, experiments were validated by testing the type strain *S. algae* CECT 5071 in the absence or presence of AEAs in MB buffered by 30 mM HEPES pH = 7.5, which minimized pH oscillations during 24-h static incubation (Supplementary Fig. 4a). The corresponding results were similar to those recorded in unbuffered medium (Supplementary Fig. 4b) showing that AEA-induced changes in biofilm formation are not caused by pH changes. Taken together, these results indicate that DMSO and nitrate-induced biofilm formation is a strain-specific phenotype independent of pH.

### DMSO respiration is not conserved in *S. algae*

**Fig. 2:**
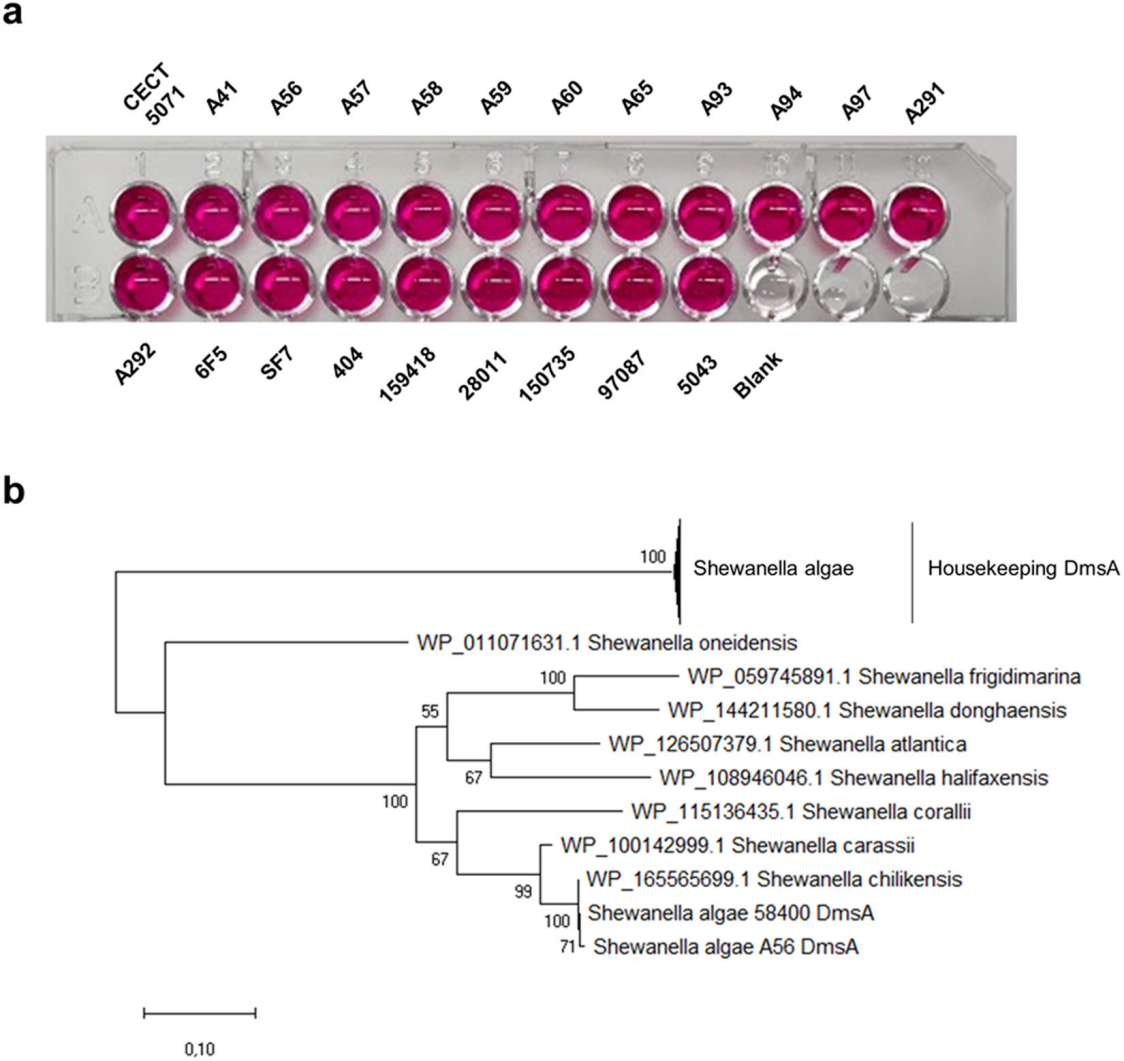
Nitrate and DMSO reduction capacity of *S. algae* strains. **a** Biofilm induction is not related to the ability of the strains to utilize the supplemented compound, as indicated by a qualitative nitrate reduction test performed for all 21 strains, showing nitrite production as indicated by the formation of a pink complex. Uninoculated MB medium supplemented with 35 mM nitrate was used as blank. **b** Evolutionary history of DmsA orthologs inferred by using the Maximum Likelihood method and Whelan and Goldman model. Bootstrap support values are indicated in the nodes of the phylogenetic reconstruction.

We hypothesized that respiration of the supplemented compounds triggered the biofilm formation response. However, we wondered whether the respiratory activity differed for strains that responded with enhanced biofilm formation. To answer this question, we qualitatively assessed nitrate reductase activity in *S*. *algae* strains by measuring the accumulation of nitrite in static cultures supplemented with 35 mM nitrate. A straightforward colorimetric assay with endpoint determination of nitrate reductase activity in live bacteria^20^ showed similar overall nitrate reductase activity in all strains (Fig. 2a). This indicates that the biofilm formation response is not related to an intrinsic capacity of a given strain to reduce the supplemented AEA.

The genomes of these and other *S. algae* strains have been sequenced as part of ongoing comparative genomics studies (Martín-Rodríguez et al., unpublished). In *S*. *algae*, nitrate reduction occurs in the periplasm and relies in a duo-NAP system, comprised of NAP-α (encoded by the *napEDABC* operon) and NAP-β (encoded by the *napDAGHB* operon), being NapA-α and NapA-β the major catalytic subunits, respectively. To investigate genomic determinants for the observed biofilm phenotypes, BLASTp searches were conducted to identify CECT 5071 NapA-α and NapA-β orthologs in the *S. algae* strains used. Both components of the NAP system were present in all *S. algae* genomes analyzed, with amino acid sequence identities between 97.1–99.9% for NapA-α and 98.1–99-1% for NapA-β with respect to the corresponding protein in the type strain (data not shown), thus indicating that the two nitrate reductases are conserved across *S. algae* isolates.

The terminal DMSO reductase DmsEFABGH is involved in extracellular DMSO respiration and uses several sulfoxides and *N*-oxides as substrates^21,22^, with DmsA being the major catalytic subunit. We next performed BLASTp searches of *S. algae* CECT 5071 DmsA orthologs to identify the presence of DMSO reductases in our sequenced *S. algae* genomes. We could retrieve hits for all *S*. *algae* strains except for A41, A57, and 97087. Genes predicted to encode the *dmsEFABGH* operon are located between *accB* and *rpmE* on the *S. algae* CECT 5071 chromosome. Inspection of the WGS of strains *S. algae* A41, *S. algae* A57, and *S. algae* 97087 revealed absence of this operon (data not shown). Besides, DmsA orthologs with a significantly lower sequence identity were found in *S. algae* A56 and *S. algae* CCUG 58400 (not used in this study, originally described as *Shewanella upenei*^23^ but demonstrated to be a later heterotypic synonym of *S. algae*^24^) that shared only modest sequence identity (55.8% both) with the reference protein. Interestingly, their sequence was almost identical to the DmsA homolog of *S. chilikensis* (GenBank accession: WP_165565699.1). A maximum-likelihood reconstruction of the evolutionary relationships of *S. algae* DmsA in relation with other *Shewanella* spp. DmsA orthologs is presented in Fig. 2b. DmsA orthologs from *S. algae* A56 and *S. algae* CCUG 58400 are within a well-supported separate clade, that is only distantly related to the housekeeping *S. algae* DmsA. Altogether, these findings suggest DMSO respiration is a variable, potentially adaptive feature in *S. algae*, with evidence of selective loss or acquisition from other *Shewanella* spp.

### Surface-associated electron acceptors enhance *S. algae* substrate colonization

**Fig. 3:**
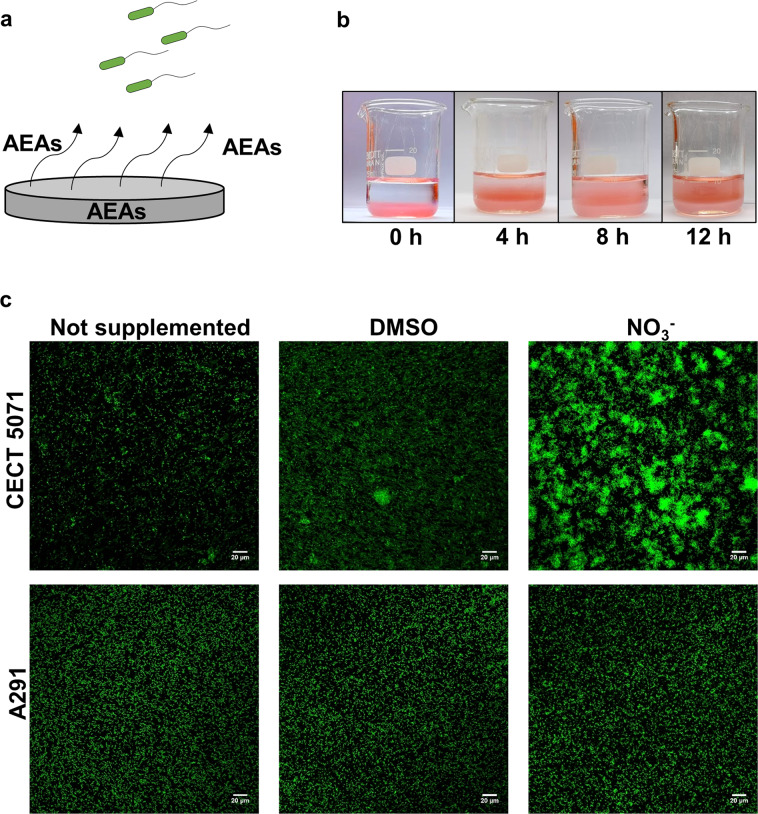
Electron acceptors promote strain-specific biofilm formation of *S. algae*. **a** Experimental set-up to assess biofilm formation by *S. algae*. Alternative electron acceptors (AEAs) were incorporated into ultra-pure agarose, from which they are progressively released. **b** Visualization of AEA release from agarose. Shown is a recipient with agarose containing cobalt (II) nitrate that is overlaid with water. **c** Surface colonization patterns of *S. algae* CECT 5071 and *S. algae* A291 on agarose plugs containing either no AEAs or 35 mM of the selected AEAs DMSO or sodium nitrate visualized by Confocal Laser Scanning Microscopy after staining with the BacLight viability kit. Representative images are shown.

Since *S. algae* encounters diverse AEAs in its native ecosystems, we hypothesized that biofilm formation could be a selective response promoting bacterial colonization of distinct substrata that are abundant in AEAs, a process that could lead to differential ecological niche occupation by distinct isolates. To test this hypothesis, we selected DMSO and nitrate as two representative AEAs and two *S. algae* strains, the DMSO and nitrate responder *S. algae* CECT 5071 and the non-responder *S. algae* A291. To mimic an environmentally relevant situation, the AEAs were incorporated into an agarose pad (Fig. 3a), and biofilm formation of each strain was examined using non-supplemented agarose as a control. The release of immobilized AEAs from the solid agarose pad could be visualized using the colored cobalt (II) nitrate as illustrated in Fig. 3b. *S. algae* CECT 5071 exhibited substantially increased biofilm formation on the agarose surface upon incorporation of DMSO or nitrate (Fig. 3c), as observed with supplemented medium. Consistently, while a monolayer of cells was observed in non-supplemented agarose, elaborated biofilm structures were observed primarily in the presence of nitrate and to a lesser degree with DMSO. Conversely, non-responsive *S. algae* A291 showed a similar low biofilm formation compared to the non-supplemented agarose pad irrespective of whether DMSO or nitrate had been added to the agarose (Fig. 3c). Thus, induction of biofilm formation is preserved upon incorporation of soluble AEAs into an agarose pad and their gradual release from a surface. This experimental set-up mimics environmentally relevant situations *S. algae* may find, for example, when bacteria encounter decaying organic matter in the aquatic milieu or on the surface of organisms such as fish or algae.

### Respiration and biofilm formation are intimately related in *S. algae*

**Fig. 4:**
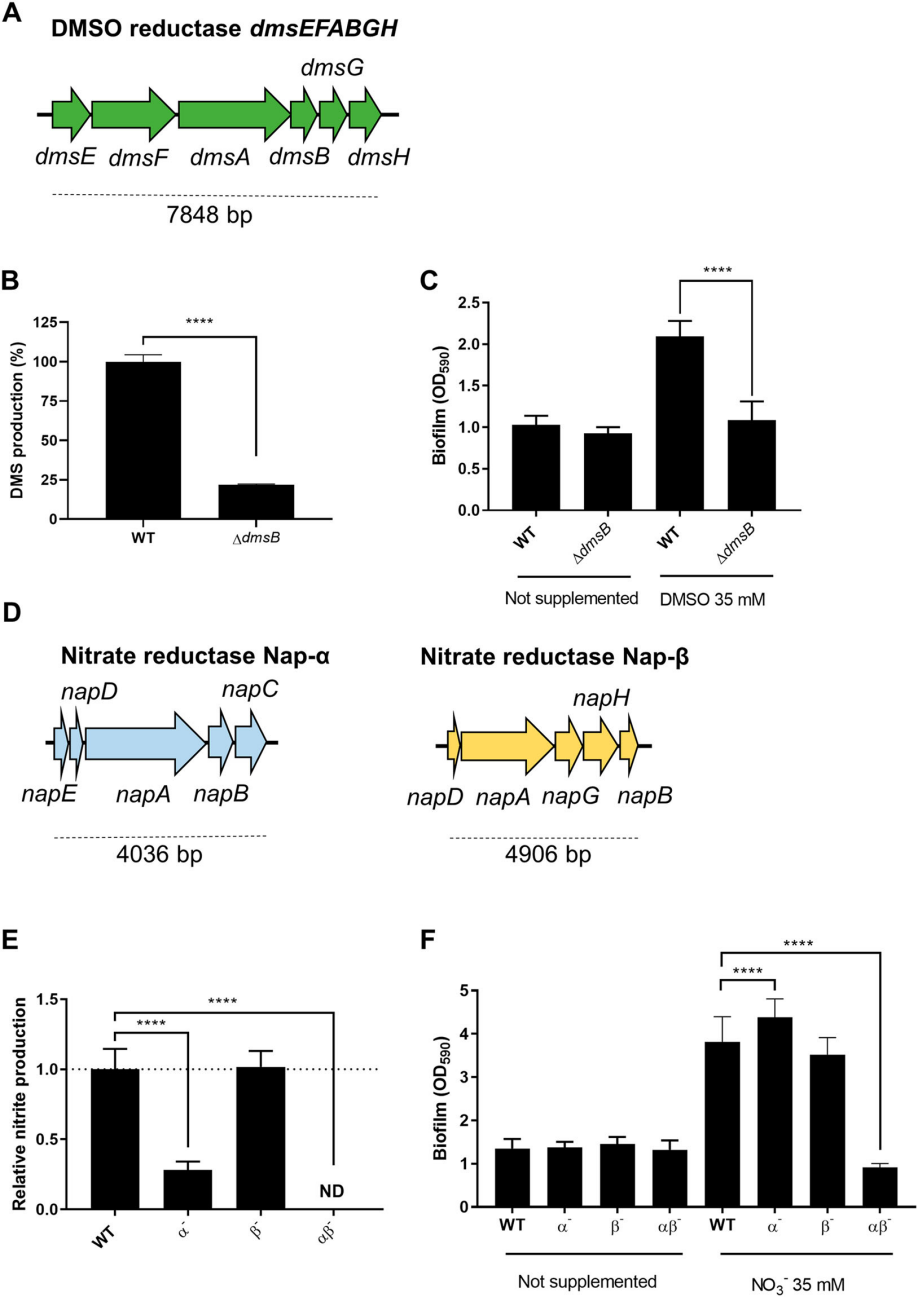
Disruption of terminal reductase activity abrogates the biofilm response. **a** Schematic of the DMSO reductase encoding *dmsEFABGH* operon of *Shewanella algae*. **b** DMSO reduction of the WT strain and *dmsB* in-frame deletion mutant (****P < 0.0001, two-tailed unpaired *t* test) as evidenced by measuring DMS production of three independent cultures supplemented with 35 mM DMSO. **c** Biofilm formation of *S. algae* CECT 5071 WT and ∆*dmsB* in the absence or presence of DMSO (****P < 0.0001, two-tailed unpaired *t* test). Data represent the average and SD of three biological replicates with seven technical replicates each. **d** Schematic of the periplasmic nitrate reductase NAP-α encoding operon *napEDABC* and NAP-β encoding operon *napDAGHB* of *S. algae*. **e** Nitrate reduction by the single mutants ∆*napABC* (α^−^) and ∆*napA* (β^−^), and the double mutant ∆*napABC* ∆*napA* (αβ^−^) as determined by nitrite accumulation with respect to the WT strain upon supplementation with 35 mM nitrate (****P < 0.0001; one-way ANOVA followed by Dunnett’s post-hoc test; ND no nitrite production detected). Data represent the average and SD of three biological replicates with four technical replicates per sample. **f** Biofilm formation of *S. algae* CECT 5071 WT and nitrate reduction mutants in the absence or presence of sodium nitrate (****P < 0.0001, one-way ANOVA followed by Dunnett’s post-hoc test).

To investigate the physiological basis of AEA-induced biofilm formation in *S. algae* CECT 5071, we next questioned whether respiration of the supplemented AEA is required for biofilm formation. To provide experimental evidence of an association between biofilm formation and reduction of AEAs, we first deleted the *dmsB* gene encoding the DMSO reductase subunit B of the DmsEFABGH reductase (Fig. 4a). To assess DMSO reduction by the WT strain and its Δ*dmsB* mutant derivative, we quantified the generation of dimethylsulfide (DMS), the product of DMSO respiration, in static cultures supplemented with 35 mM DMSO. Deletion of *dmsB* impaired the capacity to reduce DMSO by >75% (Fig. 4b) as determined by GC-MS.

We next studied biofilm formation in static cultures of the WT and mutant strain in MB in the absence or presence of 35 mM DMSO. In the absence of DMSO, both the WT strain and its Δ*dmsB* mutant exhibited similar biofilm formation and growth patterns; the latter characterized by preferential growth as a pellicle at the air–liquid interface (Fig. 4c and Supplementary Fig. 5a). In the presence of DMSO, WT growth was stimulated as compared to the non-supplemented control and occurred at the air–liquid interface as well as in the lower layers of the well. In contrast, growth of the ∆*dmsB* mutant in the presence of DMSO was similar to that in non-supplemented MB (Supplementary Fig. 5a). Biofilm formation in the WT was increased ~2-fold with respect to non-supplemented cultures as previously observed (Fig. 4c), an increase caused by higher biofilm formation on the bottom of the well (Supplementary Fig. 5a), whereas the Δ*dmsB* mutant generated a similar amount of biofilm compared to the control without DMSO (Fig. 4c and Supplementary Fig. 5a), indicating that DMSO respiration is required for the increase in relative biofilm formation upon supplementation with DMSO.

In *S. algae* CECT 5071 supplementation of MB with nitrate caused a more pronounced increase in biofilm formation than supplementation with DMSO (Fig. 3c). To extend the hypothesis of respiration-driven biofilm formation in *S. algae*, we deleted the nitrate reductase subunits A-C (*napABC*) from NAP-α, and the nitrate reductase subunit A (*napA*) from NAP-β (Fig. 4d) in the CECT 5071 genetic background that resulted in the single in-frame deletion mutants Δ*napABC* (hereafter referred to as α^−^) and Δ*napA* (hereafter referred to as β^−^), and the double deletion mutant Δ*napABC* Δ*napA* (hereafter referred to as αβ^−^). To assess the capacity of the WT versus the mutant strains to respire nitrate, we measured the production of nitrite, the product of nitrate reduction, in static cultures supplemented with 35 mM nitrate. Deletion of NAP-α genes reduced nitrite accumulation by almost 80%, whereas deletion of NAP-β genes did not significantly affect nitrate reduction in comparison to the WT (Fig. 4e). Thus, the catalytic activity of NAP-α represents the major fraction of the nitrate reduction capacity compared to NAP-β under the assay conditions used for *S*. *algae* CECT 5071. Deletion of *napABC* and *napA* rendered *S. algae* unable to produce nitrite upon addition of nitrate (Fig. 4e).

We next analyzed biofilm formation of the mutants in static cultures with and without 35 mM nitrate. In the absence of nitrate, the WT and all mutants formed a similar amount of biofilm (Fig. 4f), and the patterns of growth and biofilm formation on the walls and bottom of the wells were also similar (Supplementary Fig. 5b). In the presence of nitrate, growth in the anoxic layers of the wells was observed for the WT and mutants lacking a functional NAP-α or NAP-β, but not in the nitrate reduction null mutant αβ^−^ (Supplementary Fig. 5b). While biofilm formation in the β^−^ mutant was not significantly different from that of the WT strain, the α^−^ mutant formed significantly more biofilm than the WT (Fig. 4f), an increase that was due to higher biofilm formation on the bottom of the well as visually determined by CV staining (Supplementary Fig. 5b). In contrast, the double mutant αβ^−^, unable to reduce nitrate, did not respond to nitrate supplementation with increased biofilm formation with respect to non-supplemented cultures (Fig. 4f and Supplementary Fig. 5b). This indicates that, in analogy to DMSO, nitrate respiration drives biofilm formation in *S. algae* CECT 5071, and suggests that each nitrate reductase plays a distinct role on biofilm formation upon nitrate addition.

### Reductase activity is required to elicit biofilm formation

So far, our genetic analyses demonstrate that respiration of AEAs can selectively act as driver for biofilm formation. In WT *S. algae* CECT 5071, AEA-induced biofilm formation is dose-dependent, as shown by dose-response measurements up to a DMSO or nitrate concentration of 70 mM (Supplementary Fig. 6). These experiments also showed that the onset of AEA mediated stimulation of biofilm formation was at ~4 mM for nitrate and 8 mM for DMSO. The Δ*dmsB* mutant did not respond with enhanced biofilm formation upon addition of DMSO. In contrast, single mutants of nitrate reductases NAP-α (Δ*napABC*) and NAP-β (Δ*napA*) showed a dose-dependent response. As expected, the αβ^−^ mutant did not respond to nitrate.

**Fig. 5:**
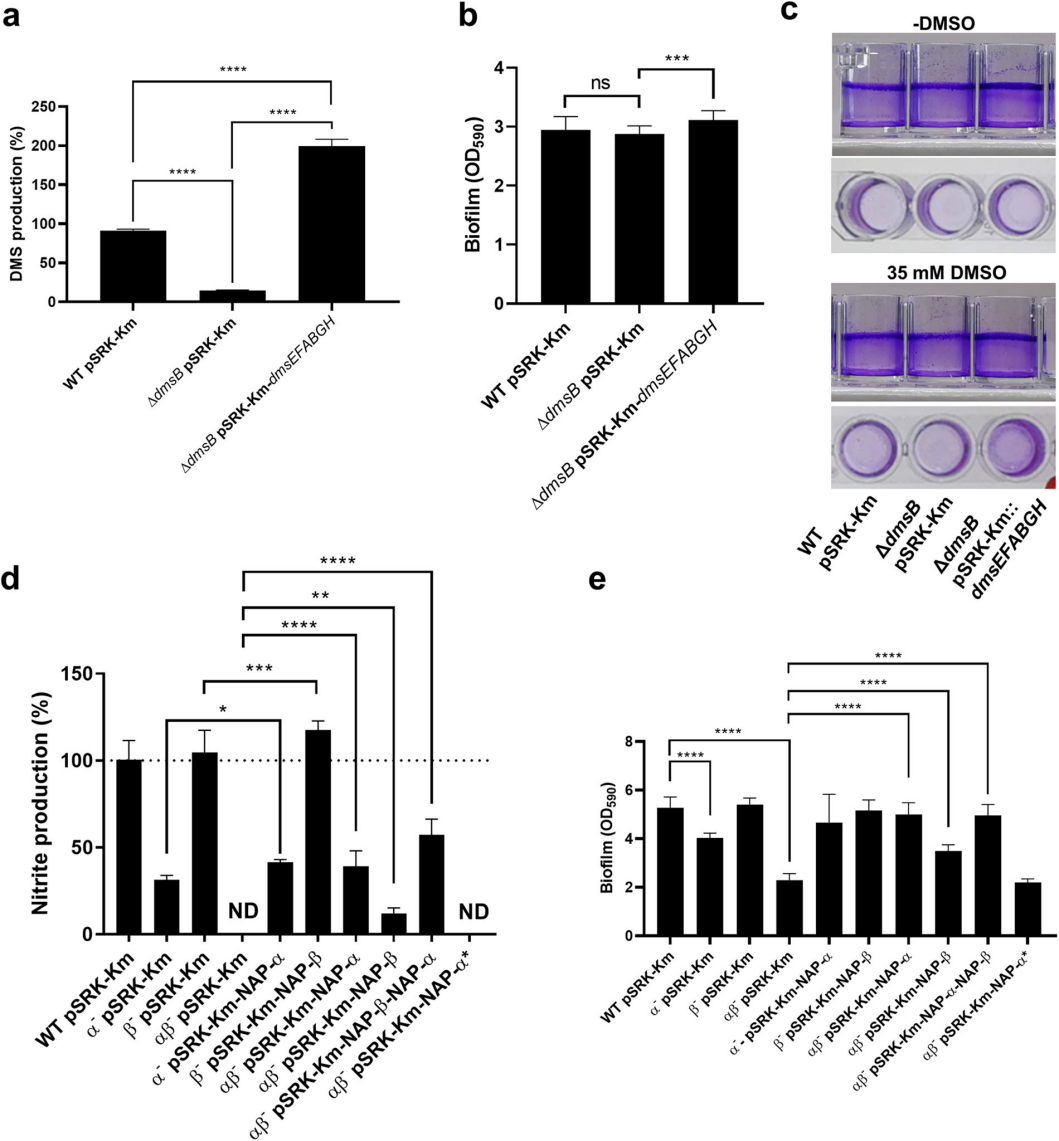
Ectopic expression of terminal reductases restores catalytic activity and biofilm formation. **a** Restoration of DMSO reductase activity as determined by DMS production in the ∆*dmsB* mutant upon overexpression of the *dmsEFAGHB* operon (average ± SD, *n* = 3) in comparison to the WT and ∆*dmsB* strains harboring the empty expression plasmid pSRK-Km (****P < 0.0001; one-way ANOVA followed by Tukey’s post-hoc test). **b** Biofilm formation of the complemented ∆*dmsB* mutant and the corresponding WT and ∆*dmsB* empty pSRK-Km vector controls. Data represent the average and SD of three biological replicates with seven technical replicates per sample. Statistical significance of the differences was determined by one-way ANOVA followed by Tukey’s post-hoc test (***P < 0.001, ns not significant). **c** Biofilm formation patterns on the walls and bottom of the wells of *S. algae* WT pSRK-Km, *S. algae* ∆*dmsB* pSRK-Km, and complemented *S. algae* ∆*dmsB* pSRK-Km-*dmsEFABGH* as visually assessed by CV staining. Note that biofilm formation differences on the bottom of the wells by the WT harboring the empty plasmid pSRK-Km and complemented mutant with respect to the ∆*dmsB* mutant harboring the empty expression vector are not apparent from the quantitative determinations shown on **b** because of the vector effect. **d** Nitrate reductase activity in complemented α^−^, β^−^, and αβ^−^ mutants as determined by nitrite production, as well as in a catalytic *napEDABC* mutant in which three of the four cysteines of the 4Fe-4S cluster of NapA had been replaced by serine. Data represent the average and SD of three biological replicates with four technical replicates per sample. Statistical significance of the differences was determined by one-way ANOVA followed by Tukey’s post-hoc test (*P < 0.05, **P < 0.01, ***P < 0.001, ****P < 0.0001, ND no nitrite production detected). **e** Biofilm formation of complemented nitrate reduction mutants and the corresponding WT and nitrate reduction null strains harboring the empty pSRK-Km plasmid, and the catalytic mutant. Data represent the average and SD of three biological replicates with seven technical replicates each. Statistical significance of the differences was determined by one-way ANOVA followed by Tukey’s post-hoc test.

To demonstrate that the mutant phenotypes were specific for the deletion of reductase subunits and not a consequence of secondary mutations, we complemented the ∆*dmsB* mutant with the *dmsEFABGH* operon cloned under the *lac* promoter in the pSRK-Km vector. In the complemented Δ*dmsB* mutant, DMS production was duplicated with respect to WT levels (Fig. 5a and Supplementary Fig. 7), compatible with overexpression of the reductase upon β-d-1-thiogalactopyranoside (IPTG) induction. The introduction of the empty pSRK-Km plasmid in the WT or Δ*dmsB* strains did not significantly change DMS production with respect to the corresponding parental strains (data not shown). Introduction of the empty vector pSRK-Km per se increased biofilm formation, leading to a higher baseline level of biofilm formation (Fig. 5b and Supplementary Fig. 8) that masked the effect of DMSO addition. While no significant differences were observed between biofilm levels of the WT and ∆*dmsB* harboring the empty plasmid, the complemented mutant though showed increased biofilm formation (Fig. 5b). This could also be qualitatively assessed visually as the WT and ∆*dmsB* pSRK-Km::*dmsEFABGH* strains tended to form more biofilm on the bottom of the wells in the presence of DMSO, although the effect of the electron acceptor was negligible compared to the high level of biofilm formation at the air–liquid interface caused by plasmid introduction (Fig. 5c).

To complement the nitrate reductase mutants α^−^, β^−^, and αβ^−^, individual *napEDABC* and *napDAGHB* operons and both operons in tandem were cloned under the *lac* promoter into the pSRK-Km vector. Nitrate reductase activity was partially restored to ~40% by ectopic expression of *napEDABC* in the α^−^ mutant with low levels of nitrate reductase (Fig. 5d). Nitrate reductase activity of the β^−^ mutant, which presented a nitrate reduction capacity similar to the WT, was significantly increased upon expression of *napDAGHB*. Complementation of the αβ^−^ strain with *napDAGHB* only marginally restored nitrate reductase activity, whereas expression of *napEDABC*, or both *napDAGHB* and *napEDABC*, restored nitrate reductase activity to ~40 and 60% of the WT levels, respectively. As the induction of biofilm formation caused by nitrate is higher than that caused by DMSO, the effect of nitrate supplementation was not abrogated by plasmid introduction. Thus, complemented α^−^ and β^−^ mutants showed similar biofilm formation phenotypes than the WT strain upon nitrate supplementation (Fig. 5e). Partial complementation of the WT biofilm phenotype upon nitrate addition was recorded for the αβ^−^ pSRKKm-NAP-β strain, whereas levels comparable to the WT were recorded for the αβ^−^ pSRKKm-NAP-α and αβ^−^ pSRKKm-NAP-α-NAP-β strains (Fig. 5e). Altogether, these data indicate that restoration of reductase activity restores the biofilm formation response in the presence of the electron acceptors.

To demonstrate the specificity of our results, we engineered a *napEDABC* operon containing a catalytically inactive NAP-α reductase by replacing three of the four cysteines, Cys48, Cys51, and Cys55 responsible for coordination of the 4Fe-4S cluster of NapA, with serine. Overexpression of the resulting mutant NAP-α* in the pSRK-Km plasmid in the αβ^−^ background did not restore nitrite production (Fig. 5d), and biofilm formation remained at the αβ^−^ mutant levels upon supplementation with nitrate (Fig. 5e). This demonstrates that the reductase activity, and not the mere expression of the reductase per se in the periplasm, triggers the biofilm formation response.

### Global transcriptional changes induced by alternative electron acceptors

**Fig. 6:**
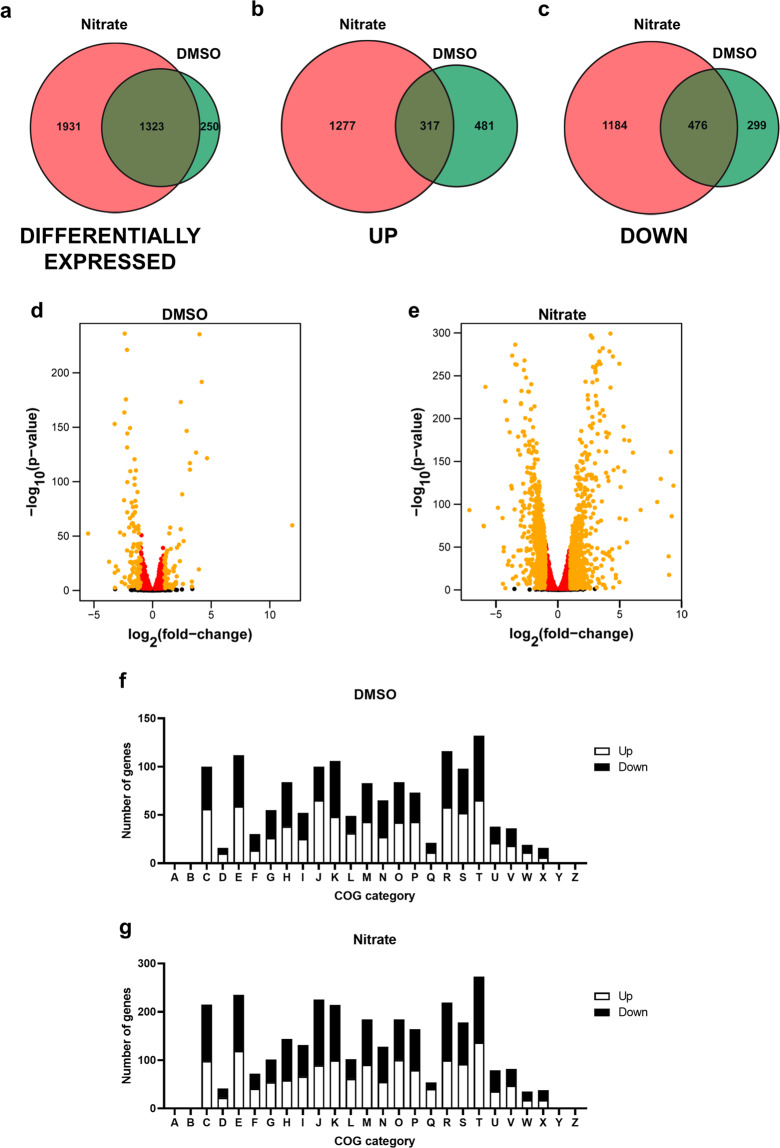
Transcriptomic profiles of *S. algae* CECT 5071 static cultures supplemented with DMSO or nitrate. Venn diagrams of (**a)** differentially expressed genes (P-adj < 0.05) in both treatments, (**b)** genes with upregulated transcript levels and (**c)** genes with downregulated transcript levels. Volcano plots showing alterations in gene transcript levels in static cultures supplemented with 35 mM DMSO (**d**) and nitrate (**e**). In red are the genes that are significantly differentially regulated (P-adj < 0.05) with a fold-change value below 2 or above 0.5. In orange are the rest of differentially expressed genes. Black indicates genes not significantly differentially regulated. COG assignation of differentially expressed genes in *S. algae* CECT 5071 upon addition of 35 mM DMSO (**f**) or sodium nitrate (**g**) versus control cultures without electron acceptor addition. COG categories: [A] RNA processing and modification; [B] Chromatin structure and dynamics; [C] Energy production and conversion; [D] Cell cycle control, cell division, chromosome partitioning; [E] Amino acid transport and metabolism; [F] Nucleotide transport and metabolism; [G] Carbohydrate transport and metabolism; [H] Coenzyme transport and metabolism; [I] Lipid transport and metabolism; [J] Translation, ribosomal structure and biogenesis; [K] Transcription; [L] Replication, recombination and repair; [M] Cell wall/membrane/envelope biogenesis; [N] Cell motility; [O] Post-translational modification, protein turnover, and chaperones; [P] Inorganic ion transport and metabolism; [Q] Secondary metabolites biosynthesis, transport, and catabolism; [R] General function prediction only; [S] Function unknown; [T] Signal transduction mechanisms; [U] Intracellular trafficking, secretion, and vesicular transport; [V] Defense mechanisms; [W] Extracellular structures; [Y] Nuclear structure; and [Z] Cytoskeleton.

To gain a deeper understanding of the physiological responses induced by different AEAs, transcriptional profiles were obtained from *S. algae* CECT 5071 grown in static culture in the absence or presence of 35 mM DMSO or nitrate (Supplementary Data 1). Statistically significant changes in transcript levels (P-adj ≤ 0.05) of a total of 3553 genes were noted in *S. algae* CECT 5071 exposed to DMSO or nitrate (Fig. 6a). There was a tendency between the number of transcripts changed in the presence of a given AEA and the corresponding effect on biofilm formation, since more genes had altered transcript levels in the presence of nitrate as compared to DMSO. Thus, there were 1573 genes with altered transcript levels in the presence of DMSO (Fig. 6b–d; 798 upregulated and 775 downregulated) and 3254 in the presence of nitrate (Fig. 6b, c, e; 1594 upregulated and 1660 downregulated). There was a substantial overlap in the transcriptional response to both AEAs, with 1323 genes having altered transcript levels (either upregulated or downregulated) in either condition (Fig. 6a).

Next, genes were classified according to functionality in clusters of orthologous genes (COGs). In the DMSO response, the highest number of upregulated and downregulated transcripts (132 genes, 65 upregulated, and 67 downregulated) belonged to the COG category signal transduction mechanisms [T] (Fig. 6f). This was also the case for nitrate, that modulated transcript levels of 273 genes belonging to this category (136 upregulated and 137 downregulated, Fig. 6g). These data suggest that respiration of AEAs involves downstream intracellular signaling events resulting in increased biofilm formation, consistent with the transcriptional landscape described above. An enrichment analysis using PANTHER^25^ showed a significant enrichment (*P* < 0.05) for the ‘sulfate assimilation’ and ‘de novo purine biosynthesis’ categories for both treatments, although the high number of transcripts showing altered expression levels in the presence of DMSO and nitrate suggest that both electron acceptors elicit global physiological changes. We also classified upregulated and downregulated transcripts for each condition into gene ontology (GO) categories. GO terms of the categories “biological processes” and “molecular function” are presented for DMSO (Supplementary Fig. 9a, b) and nitrate (Supplementary Fig. 9c, d). In both treatments, the most populated GO terms under “biological processes” were metabolic processes (GO:0008152) and cellular processes (GO:0009987), irrespective of the directionality of the expression change, indicative of a major physiological reprogramming imposed by AEA supplementation. Likewise, “catalytic activity” (GO:0003824) was the most represented GO term of the “molecular function” category for both DMSO and nitrate supplementation, which is also consistent with the notion that both compounds induce a physiological re-adaptation.

### A putative role of c-di-GMP signaling

**Fig. 7:**
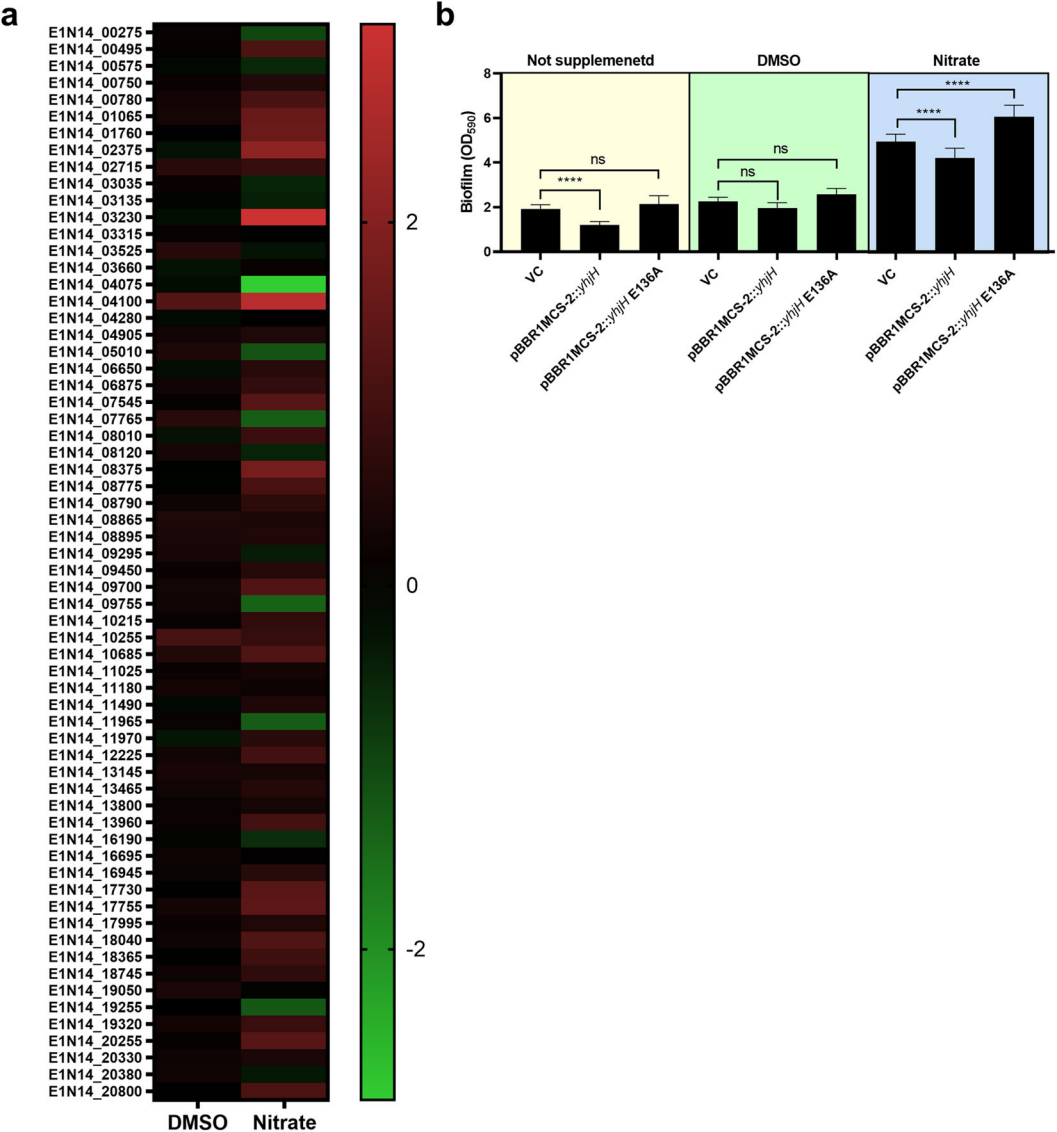
Putative role of c-di-GMP signaling on AEA-induced biofilm formation. **a** Heatmap showing transcriptional changes (log_2_ fold-change) of genes encoding putative c-di-GMP turnover proteins upon DMSO or nitrate supplementation. **b** Biofilm formation of *S. algae* CECT 5071 WT harboring the empty plasmid pBBR1MCS-2 (denoted as VC for “vector control”) or expressing the PDE from *S*. Typhimurium YhjH or the catalytically inactive derivative YhjH E136A cloned in this plasmid in the absence or in the presence of 35 mM DMSO or nitrate. Data represent the average and SD of three biological replicates with seven technical replicates per sample. Statistical significance of the differences was determined by one-way ANOVA followed by Tukey’s post-hoc test (****P < 0.0001, ns not significant).

As noted above, signal transduction mechanisms were highly affected by DMSO or nitrate supplementation. A hallmark of the genus *Shewanella* is the abundance of proteins that catalyze the synthesis or breakdown of c-di-GMP, suggesting a central role of c-di-GMP-mediated signaling in *Shewanella* biology. We identified 63 putative c-di-GMP turnover proteins in the genome of *S. algae* CECT 5071 as part of ongoing genomic studies (Martín-Rodríguez et al., in preparation). In the absence of specific gene nomenclature, we refer to the genes encoding these proteins with provisional generic names, but the corresponding GenBank locus tags are given in parentheses to unambiguously identify the genes. To investigate their transcriptional changes induced by DMSO or nitrate supplementation, we plotted the transcriptome of the 63 genes encoding putative diguanylate cyclases (DGCs) and c-di-GMP phosphodiesterases (PDEs) containing GGDEF/EAL/HD-GYP domains in a heatmap (Fig. 7a). This analysis revealed discrete changes caused by DMSO addition, with gene *WT_00831* (E1N14_04100) encoding a PAS-GGDEF domain containing protein being the most differentially upregulated by 2.4-fold with respect to the control without electron acceptor supplementation. Nitrate supplementation caused more extensive changes in the c-di-GMP turnover transcriptome, with the putative DGCs *WT_00655* (E1N14_03230) and *WT_00831* (E1N14_04100) being the most differentially upregulated by 8.5- and 6.8-fold, respectively, with respect to control cultures. Another gene encoding a GGDEF-domain protein, *WT_00826* (E1N14_04075) was the most downregulated, 0.14-fold, with respect to the control. Altogether, these data support the notion of an involvement of c-di-GMP signaling in respiration-mediated regulation of biofilm formation in *S. algae* CECT 5071.

We reasoned that if c-di-GMP signaling mediates AEA-dependent biofilm formation, a decrease of intracellular c-di-GMP pools would revert the phenotype upon AEA supplementation. To artificially alter c-di-GMP levels, the heterologous c-di-GMP PDE YhjH from *S.* Typhimurium and the catalytically inactive derivative YhjH E136A were cloned into plasmid pBBR1MCS-2, introduced in *S. algae* CECT 5071, and the biofilm phenotypes of YhjH and YhjH E136A expressing strains were compared with respect to that of the control strain harboring the empty vector in plain MB and MB supplemented with 35 mM DMSO or nitrate (Fig. 7b). Expression of YhjH significantly decreased biofilm formation in MB, whereas biofilm formation of the strain expressing YhjH E136A was not significantly different than that of the vector control. In the presence of DMSO there were no statistically significant differences between the vector control and the YhjH or YhjH E136A expressing strains, although a trend towards lower biofilm formation was noted in the strain expressing the active PDE. In the presence of nitrate, YhjH expressing cells formed significantly lower biofilm than the vector control whereas cells expressing YhjH E136A formed statistically significantly higher biofilm. Taken together, these results suggest that a decrease of intracellular [c-di-GMP] decreases biofilm formation in static culture and counteracts to some extent the effect of AEA supplementation.

Finally, to validate RNA-seq results and to test the reproducibility of our data, RNA was isolated from static *S. algae* CECT 5071 cultures as performed for transcriptomic analysis, and the expression of six relevant genes was tested by qRT-PCR and compared to transcript levels obtained by RNA-seq: *dmsB* (E1N14_09855); *napA*-α (E1N14_17675); *napA*-β (E1N14_03175); *WT_00826* (E1N14_04075); *WT_00831* (E1N14_04100); and *WT_00655* (E1N14_03230) (Supplementary Fig. 10). The differential expression of these genes was similar in the transcriptomic and qRT-PCR analyses, which validates the RNA-seq data.

## Discussion

In this study we demonstrate a specific differential induction of biofilm formation in response to respiration in *S. algae*. We show that the presence and subsequent utilization of soluble electron acceptors in a seawater-mimicking growth medium induces biofilm formation in some but not all *S. algae* strains tested. Disruption of the corresponding respiratory pathway as well as mutant complementation with a catalytically inactive variant abolished the capacity of DMSO or nitrate to stimulate biofilm formation.

In *S. algae*, biofilm formation is strain-specifically upregulated upon supplementation with various electron acceptors. Thereby, respiration and biofilm formation are directly coupled as mutants in the respective terminal reductase did not respond with altered biofilm formation. A physiological link between cellular respiration and regulation of biofilm formation has been observed in other bacteria. In *Pseudomonas aeruginosa*, nitrate respiration has been found to contribute to biofilm formation and development under static growth^26^, while in *Burkholderia pseudomallei*, nitrate respiration was found to inhibit biofilm formation by decreasing global c-di-GMP cellular pools^27^. Anaerobic respiration has been reported to induce biofilm formation in *Vibrio cholerae* and *Staphylococcus epidermidis*, thereby promoting host colonization^28,29^. In *Staphylococcus aureus*, biofilm formation is promoted under anoxic conditions in response to the oxidation state of the membrane quinone pool^30^. Opposite roles for oxygen have been reported for other *Shewanella* spp. where low oxygen promoted c-di-GMP-mediated biofilm formation in *S. putrefaciens* CN32^11^ or induced biofilm detachment in *S. oneidensis* MR-1^12^. Altogether, these findings suggest the existence of an extensive, but variable interplay between biofilm formation and the nature of the electron acceptor in respiration.

In our study we used two representative electron acceptors, nitrate and DMSO. Our results implicate that the two periplasmic nitrate reductases of *S. algae* play different, non-redundant roles under nitrate-rich conditions. Thus, a mutant lacking a functional NAP-β does not show alterations in biofilm formation and global nitrate reductase activity in comparison to the WT as determined by nitrite assessment in culture supernatants, but a mutant lacking a functional NAP-α exhibits a strong impairment in nitrate reduction capacity and altered biofilm formation patterns. The greater contribution of NAP-α to nitrate reduction under static growth conditions is also reflected by a higher expression of this reductase than the other isoform, NAP-β. The nitrate reductase is a complex multidomain protein consisting of the core subunits NapA and NapB, the chaperone NapD and variable accessory subunits^31^. NapA is the catalytic subunit containing a [4Fe-4S] cluster, whereas NapB shuttles electrons from the membrane electron transport protein CymA to NapA. Replacement of NapA-α Cys48, Cys51, and Cys55 involved in the coordination of the [4Fe-4S] cluster^32^ with serine was shown to prevent nitrate reduction and we show that a nitrate reduction-null mutant expressing such a catalytically deficient NapA-α is unable to induce biofilm formation upon nitrate supplementation. To the best of our knowledge, our work is the first to functionally analyze nitrate reductase activity (and their contribution to biofilm formation) in a *Shewanella* species harboring both NAP isoforms, as is the case for most shewanellaceae as shown by whole-genome sequencing^31^, thus establishing a functional link between respiration and biofilm formation, two prima facie independent processes.

Like nitrate, DMSO also elicited strain-specific biofilm formation. Remarkably, genome sequence analysis of *S. algae* strains showed the DMSO reductase DmsEFABGH not to be conserved across the sequenced isolates, and in some cases, not present at all. In some *S. algae* isolates, high sequence identity of the catalytic subunit DmsA with orthologs from other *Shewanella* spp. was noted and demonstrated by phylogenetic analysis. The diversity of gene clusters encoding DMSO reductases in different *Shewanella* species has been shown^22^. However, the intra-species variability of the DMSO reductase operon indicates that DMSO respiration is under strong evolutionary pressure in *S. algae*. Loss or lateral acquisition of respiratory features by *S. algae* from other *Shewanella* spp. points to currently unexplored ecological and physiological advantages.

The ability to reduce a given electron acceptor was not necessarily associated with increased biofilm formation. As illustrated for nitrate, while all *S. algae* isolates reduced nitrate to nitrite, not all of them responded with enhanced biofilm formation indicating that AEA sensing with subsequent upregulation of biofilm formation can be functionally uncoupled from AEA respiration. Sensory domains in signal transduction systems have been reported to detect nitrate and directly couple it to enhanced biofilm formation through increased levels of c-di-GMP, a ubiquitous biofilm activator. For example, in *P. aeruginosa*, nitrate sensing by the GGDEF-EAL domain containing protein MucR via its MHYT domain induces expression of the exopolysaccharide alginate^33^. Another study showed that homologs of the nitrate-sensing two-component system NarX/NarL in *B*. *pseudomallei* is involved in the regulation of biofilm formation by potentially interfering with c-di-GMP levels^27^. Furthermore, accumulation close to insoluble electron acceptors has been observed to require chemotaxis and electron transport chain components^34,35^.

The habitat of *S. algae* includes coastal and oceanic sediments as well as the water column^2^, where microbial reduction of inorganic and organic soluble and insoluble electron acceptors are a central part of the Earth’s global biogeochemical cycles^22,36,37^. DMSO is a relatively abundant compound in seawater thought to play a key role in the global sulfur cycle^38^. Most of DMSO is produced from DMS, a climate active gas responsible for the main exchange of sulfur between the oceans and the atmosphere. DMS is the main end product of the degradation of dimethylsulfoniopropionate (DMSP), an algal osmolyte synthetized by phytoplankton^39^ at an estimated rate of 10^9^ tons per year in the oceans^40,41^. As an adaptive trait, two differentially expressed *dms* operons are found in *Shewanella piezotolerans* WP3^18^. Local pools of AEAs are also available in certain host body niches colonized by *Shewanella* spp. including the intestinal tract^17^. Niche partitioning with the emergence of specific ecotypes that have lost the ability to respire on certain AEAs such as DMSO, TMAO, or thiosulfate has evolved in *S. baltica*. This type of genomic evolution is thought to have led to the existence of carbon source and redox-specialized *S*. *baltica* communities in sediments of the Baltic Sea^42,43^. In *S. algae*, niche partitioning, at least in the case of nitrate, is more complex since it combines enhanced biofilm formation with the ability to respire the AEA. Both biofilm and non-biofilm responsive strains have been found to perform dissimilatory reduction of nitrate as an AEA prototype. While the concentration of DMSO and nitrate used as a reference in this study (35 mM) is higher than that in the natural habitat, i.e. seawater, local mM concentrations of these AEAs may be found in certain niches. For example, DMSO concentrations of 30–90 mM have been reported in the coccolithophore *Emiliania huxleyi*^44^. A study has shown *S. oneidensis* to perform positive chemotaxis towards senescent *E. huxleyi* cells^45^, supporting the notion that *Shewanella* spp. is attracted by gradients of AEAs permitting their metabolization. The genera of sulfur-oxidizing bacteria *Beggiatoa* and *Thioploca* accumulate up to 500 mM nitrate intracellularly as an electron acceptor for sulfide oxidation^46^. Likewise, high nitrate concentrations could occur at the interface with nitrate-rich fecal or decaying organic material^47^.

In the natural environment, AEAs are rarely encountered homogeneously, but are present in gradients that can be sensed by microorganisms. Sedimented organic material or phytoplankton are sources of nitrate and DMSO^48–50^. Solid agarose colonization upon incorporation of the AEAs DMSO and nitrate has given direct evidence that sensing and respiration promote substrate colonization by *S. algae*. Positive chemotaxis towards soluble and insoluble AEAs has been reported for *S. oneidensis* MR-1 and *S. putrefaciens*^35,51,52^ and may contribute to the elevated biofilm development on abiotic or biotic surfaces containing AEAs^53^. *Shewanella* spp. exploit surface attachment and biofilm formation in conjunction with respiration of insoluble AEAs^54,55^. However, whether surface attachment and biofilm formation play a role in the respiration of highly diffusible AEAs is poorly characterized. Electroactivity measurements revealed increases in *Shewanella* biofilm formation upon elevation of c-di-GMP levels^13^, thus enhanced biofilm formation could correlate with improved electrical transfer.

Because of their outstanding metabolic and respiratory flexibility, regulation of gene expression, including terminal reductases, is complex in *Shewanella* spp. The anaerobic reduction pathways of *Shewanella* spp. have been shown to be promiscuous, sometimes involving the simultaneous utilization of several electron acceptors^56^. Oxygen limitation has been shown to elicit swift changes in energy metabolism, cytochrome production, and formation of conductive nanowires in *S. oneidensis* MR-1^57^. Transcriptomic analyses under defined anaerobic conditions have shown that anaerobic reduction pathways, such as nitrate and Mn(IV) reduction, are deeply intertwined in *S. algae*^58,59^. There is an oxygen gradient within biofilms, which adds another layer to the complexity of oxygen-mediated regulatory processes^60^.

Following the trend observed in biofilm formation experiments, the extension of the global transcriptional changes induced on *S. algae* CECT 5071 was higher for nitrate than for DMSO, consistent with transcriptomic analyses reported for *S. oneidensis* MR-1 upon exposure to these electron acceptors^61^. Conclusively, this observation indicates fundamental metabolic differences, and perhaps biofilm characteristics, between growth conditions to accompany the combined respiration and biofilm-driven niche adaptation. Since signal transduction genes corresponded to the most populated family under both conditions, environmental sensing and regulation are likely to play key roles in conferring the enhanced biofilm phenotype. Among the signal transduction pathways of *Shewanella* spp. that regulate biofilm formation, c-di-GMP signaling occupies a prevalent position with *Shewanella* spp. having one of the largest number of c-di-GMP turnover proteins among prokaryotes^7^. Transcription of the putative DGC with an N-terminal PAS sensory domain *WT_00831* (E1N14_04100) was found to be upregulated in the presence of both DMSO and nitrate, thus constituting a primary candidate for future studies aimed at deciphering the underlying molecular mechanism of the biofilm response. Transcript levels of other two DGCs, *WT_00826* (E1N14_04075) and *WT_00655* (E1N14_03230) were found to be significantly shifted by nitrate. This suggests c-di-GMP, or another second messenger cyclic dinucleotide signaling pathway, as a candidate pathway regulating respiration-mediated biofilm formation in *S. algae*. This notion is further supported by differential biofilm formation upon expression of the heterologous PDE YhjH and its catalytic mutant YhjH E136A from *S*. Typhimurium, presumably as a consequence of artificially altered intracellular c-di-GMP pools.

In summary, in this work we have provided evidence that reductase activity is required for AEA-induced biofilm formation in *S. algae*. Deletion mutants in the different reductases characterized the physiological role of respiration in terms of activity and biofilm induction. Given the complexity of the electron transport systems and signal transduction pathways in *S. algae*, and the strain-specific nature of this phenomenon, this work provides an entry point for follow-up studies of comparative transcriptomic analyses of responsive and non-responsive *S. algae* isolates as well as extensive mutational analyses to identify and characterize specific regulatory pathways linking respiration with biofilm formation. Furthermore, we report that biofilm formation is closely interwoven with the use of different electron acceptors by *S. algae*. We show that the two NAP isoforms of *S. algae* play substantially different roles on cellular nitrate reduction and nitrate reduction-mediated biofilm formation. We also demonstrate DMSO reduction to be a variable feature in *S. algae* with evidence of loss or horizontal acquisition in certain isolates. Respiration-driven biofilm formation may therefore constitute a mechanism of niche colonization by specialized *S. algae* strains, not only in anoxic environments where certain AEAs are abundant, but also in moderately oxygenated environments by taking advantage of their broad respiratory repertoire and reducing thereby the consequences of microbial competition for oxygen. Apart from deciphering the underlying molecular mechanisms, this work provides a sound basis for future research aimed at establishing the phylogenetic spread of the phenomenon studied here.

## Methods

### Strains and growth conditions

The *Shewanella* strains used in this study (Supplementary Table 1) were routinely grown in MB or on marine agar (MA) (Difco). *Escherichia coli* strains (Supplementary Table 1) were routinely grown in Luria broth (LB) or on Luria agar (LA). When necessary, the medium was supplemented with kanamycin (50 µg ml^−1^), streptomycin (200 µg ml^−1^), IPTG (0.5 mM), and/or diaminopimelic acid (0.3 mM).

### Genetic manipulations

The R6K-origin plasmid pKNG101 was used for allelic replacement in *S. algae* CECT 5071. For in-frame deletion of the target genes *dmsB* (minor subunit of the DMSO reductase DmsAB), *napABC* (periplasmic nitrate reductase NAP-α, subunits A, B, and C) and *napA* (periplasmic nitrate reductase NAP-β, subunit A), the chromosomal regions immediately up- and down-stream the genes of interest were PCR-amplified and fused in plasmid pKNG101 using the primers and restriction sites indicated in Supplementary Table 2. To create the *dmsB* deletion, the DNA fragments comprising the 347-bp upstream and 355-bp downstream the *dmsB* gene were initially cloned into pUC18Not using the restriction sites indicated in Supplementary Table 2, and then subcloned into pKNG101 at the NotI site. The resulting plasmids were propagated in *E. coli* DH5α λpir, isolated, and electroporated into *E. coli* MFDpir from which they were mobilized into *S. algae* CECT 5071 by conjugation. Recombinants generated by single-crossover recombination were selected on LA plates containing Sm. Double crossover was induced upon re-streaking merodiploids on plates containing 10% (w/v) sucrose. In-frame gene deletion mutants were confirmed by PCR using primers flanking the recombination sites (Supplementary Table 2) followed by Sanger sequencing. To complement the mutations, the relevant operons *dmsEFABGH*, *napDAGHB* and *napEDABC* were cloned individually or in tandem under the control of the p*lac* promoter in pSRK-Km using the primers listed in Supplementary Table 2. The cloning strains *E. coli* TOP10 and NEB-5α were used for plasmid propagation. All plasmid constructs were confirmed by Sanger sequencing.

The wild-type *yhjH* gene from *Salmonella enterica* serovar Typhimurium ATCC 14028 Nal^R^ (UMR1) and the catalytically inactive derivative YhjH E136A were cloned into pBBR1MCS-2 with primers *yhjH*-F-HindIII and *yhjH*-R-XbaI (Supplementary Table 2) using *S.* Typhimurium UMR1 genomic DNA or plasmid pRGS4^62^ as templates, respectively, followed by Sanger DNA sequencing.

### Generation of catalytically inactive NapA

To generate a catalytically inactive NapA-α enzyme, three of the four cysteines coordinating the 4Fe-4S cluster in the nitrate reductase α catalytic subunit NapA were replaced by serine (C48S, C51S and C55S). Nucleotide exchanges were introduced with the Q5 site-directed mutagenesis kit (NEB) following the manufacturer’s instructions (primers in Supplementary Table 2). The nucleotide replacements were verified by Sanger sequencing.

### Biofilm formation assays

In total, 200 µl bacterial cultures were incubated statically in 96-well plates (TPP, #92096) at 30 °C for 24 h in MB in the absence or presence of 35 mM of DMSO or sodium nitrate. Concentration-dependent biofilm formation was assessed in the absence or presence of 0.55-70 mM DMSO and nitrate. Adherent cells were quantified by crystal violet (CV) staining as previously described with minor modifications^63^. Briefly, after staining adherent cells with a 0.2% (w/v) CV solution, the biofilm-associated dye was dissolved with 30% (v/v) acetic acid. Total biofilm biomass is reported as OD_590_ absorbance. Biofilm formation patterns including pellicle formation were documented using 96-well strip plates (Greiner Bio-One, #762070).

### Phylogenetic analyses

Identification of *S. algae* CECT 5071 dimethyl sulfoxide reductase DmsA and nitrate reductase NapA orthologs was performed by BLASTp against the whole-genome sequencing contigs of 41 *S. algae* strains (Martín-Rodríguez et al., unpublished) using the Uppmax computational cluster (National Genomics Infrastructure, Sweden), followed by manual inspection of the corresponding genomic loci. The evolutionary history of terminal reductases DmsA and NapA was inferred by using the Maximum Likelihood method and the Whelan and Goldman model^64^ after 1000 bootstrap replications as implemented in MEGA X^65^. The nucleotide sequences of *dmsA* orthologs are available at the NCBI GenBank (accession numbers: MT953034-MT953072).

### Confocal microscopy

To assess biofilm formation on surfaces containing AEAs, 35 mM DMSO and nitrate were added to 2.5 ml 2.5% (w/v) ultrapure agarose (Bio-Rad), which was dispensed in 6-well tissue culture plates and allowed to solidify. Non-supplemented agarose was used as a control surface. Agarose disks were overlaid with 8 ml of bacterial suspensions containing ~1 × 10^6^ CFU ml^−1^ of *S. algae* CECT 5071, a strain responding to both AEAs with increased biofilm formation, and *S. algae* A291, a strain that does not exhibit a AEA-mediated biofilm response, and then incubated statically overnight. The bacterial cultures were then removed by aspiration and the agarose disks washed three times with sterile PBS. Biofilm bacteria were stained using the BacLight viability kit (Invitrogen). Sections of ~0.5 × 0.5 cm were excised with a sterile scalpel for imaging in an Olympus FluoView FV1000 confocal microscope. Experiments involved two biological replicates with two technical replicates each, and a minimum of three sections were imaged per technical replicate.

### Gas chromatography

Determination of DMSO reductase activity in static bacterial cultures in MB supplemented with 35 mM DMSO was performed by quantification of DMS production by GC-MS (Varian 450GC-240MS). In all, 10 ml vials containing 5 ml bacterial cultures adjusted to an initial OD_600_ of 0.05 in MB medium supplemented with DMSO were sealed and incubated at 30 °C for 24 h. Controls included a culture of *S. algae* CECT 5071 WT in MB not supplemented with DMSO, as well as sterile medium supplemented with 35 mM DMSO. The cultures were pre-conditioned at 30 °C for 2 min under agitation before extraction of 0.1 ml gas using a Head Space syringe and injection at 250 °C in split mode. A DB5-MS UI (30 m × 0.25 mm × 0.25 µm) column was used with He as carrier gas at a flow rate of 1.0 ml min^–1^. The GC oven was programmed to rise from 40 °C (3 min) to 220 °C at 100 °C min^-1^ (1 min). DMS was detected by a mass spectrometer with an EI ion source running in TIC Full Scan mode (scanning m/e 32–300). Experiments involved three biological replicates.

### Determination of nitrate reductase activity

Nitrate reductase activity in static cultures of strains grown in MB supplemented with 35 mM nitrate was determined spectrophotometrically by measuring the accumulation of nitrite in supernatants upon addition of 0.02% (w/v) *N*-(1-napthyl)ethylenediamine in 95% (v/v) ethanol and 1% sulfanilamide (w/v) in 1.5 N HCl as previously described^20^. Optical readings were taken at OD_540_ in 96-well plates. Experiments involved at least three biological replicates.

### RNA isolation and sequencing

Total RNA was isolated from static overnight cultures of *S. algae* CECT 5071 in non-supplemented MB or the same medium supplemented with 35 mM DMSO or 35 mM nitrate. Prior to RNA isolation, cultures (three biological replicates per test condition) were homogenized by inverting the tubes to ensure representative sampling of the entire bacterial population. RNA purification was performed with the Direct-Zol RNA Miniprep Plus kit (Zymo Research) following the instructions of the manufacturer. Residual genomic DNA was removed by DNase treatment with the Turbo DNA-free kit (Ambion). DNA contamination was assessed with a 40 cycle PCR using the DNase-treated RNA samples as templates. The RNA quality was examined on a 2100 Bioanalyzer system (Agilent). The relative integrity (RIN) values were 9.6–10.

Prior to sequencing, rRNA was depleted from 550 ng of total RNA using Illumina Ribo-Zero rRNA removal kit for Gram-negative bacteria. Sequencing libraries were then prepared using Illumina TruSeq stranded total RNA kit. The libraries were pooled and 1% of PhiX control library was added to the pool. The pool was sequenced on a HiSeq 2500 machine in High Output run mode using v4 chemistry. The read length was PE125.

Low quality sequence reads and adapters were removed with AfterQC^66^ using default parameters and the remaining contaminating rRNA was removed with SortMeRNA^67^. Read counts per gene were obtained using the package *Salmon*^68^ with default parameters. To identify differentially expressed genes, the DESeq2 R/Bioconductor package was used^69^. RNA-seq reads were deposited at the NCBI database (accession number: GSE133627).

The annotation of *S. algae* CECT 5071 genes was classified into functional families using the Cluster of Orthologous Groups (COG) by BLASTP with 60% minimum alignment coverage, 50 minimum bitscore, and 35% minimum percent identity.

Proteins were classified into families and subfamilies with an ontology term (GO), PANTHER protein class, and PANTHER pathway using its classification system (www.pantherdb.org^25^). Statistical enrichment analysis for differentially expressed genes was considered as significant when the FDR-adjusted *P*-value was inferior to 0.05. Analyses were assisted by Python scripts.

### qRT-PCR validation of RNA-seq data

Quantitative real time PCR was used to validate differential gene transcript levels using RNA isolated from *S. algae* CECT 5071 cultures under the same conditions as described above. The primers used in qRT-PCR reactions are listed in Supplementary Table 2. Complementary DNA (cDNA) was synthesized with the QuantiTect Reverse Transcription Kit (Qiagen) using 1 μg of RNA. Quantitative PCR reactions were prepared with the Power SYBR Green PCR Master Mix (Applied Biosystems) and run on a LightCycler 480 instrument (Roche) using the pre-defined SYBR Green reaction protocol. Relative gene expression was calculated using the ΔΔCt method^70^.

### Reporting summary

Further information on experimental design is available in the Nature Research Reporting Summary linked to this paper.

## Supplementary information

Supplementary Materials

Supplementary Data 1

Reporting Summary

## Data Availability

RNA sequencing data that support the findings of this study have been deposited in the NCBI GenBank with the accession code GSE133627. The Whole Genome Shotgun project of *S. algae* CECT 5071 is available at the NCBI GenBank under the accession code SMNR00000000. The nucleotide sequences of *dmsA* orthologs used for phylogenetic analysis are available at the NCBI GenBank under accession codes MT953034-MT953072.
