# Supplementary material for: Reduction of alternative electron acceptors drives biofilm formation in Shewanella algae
Source: NPJ Biofilms Microbiomes. 2021 Jan 27;7:9. doi: 10.1038/s41522-020-00177-1 (PMC7840931; doi:10.1038/s41522-020-00177-1)
Supplement: Supplementary file 1 — Supplementary Materials [file 41522_2020_177_MOESM1_ESM.pdf]

Reduction of alternative electron acceptors drives biofilm formation in *Shewanella algae*

Alberto J. Martín-Rodríguez, José A. Reyes-Darias, David Martín-Mora, José M. González, Tino Krell and Ute Römling

SUPPLEMENTARY MATERIALS

Index of contents:

Supplementary Figures:

Supplementary Figure 1. Growth and biofilm formation patterns of *S. algae* strains in MB medium.

Supplementary Figure 2. Growth and biofilm formation patterns of *S. algae* strains in MB medium supplemented with 35 mM DMSO.

Supplementary Figure 3. Growth and biofilm formation patterns of *S. algae* strains in MB medium supplemented with 35 mM nitrate.

Supplementary Figure 4. Effect of pH changes on *S. algae* CECT 5071 biofilm formation.

Supplementary Figure 5. Growth and biofilm formation patterns of *S. algae* WT and reductase mutants.

Supplementary Figure 6. Dose-dependent biofilm formation upon alternative electron acceptor supplementation in *S. algae* CECT 5071.

Supplementary Figure 7. Dimethylsulfide (DMS) detection in *S. algae* CECT 5071 static cultures supplemented with 35 mM DMSO.

Supplementary Figure 8. Effect of plasmid pSRK-Km on *S. algae* CECT 5071 static biofilm formation.

Supplementary Figure 9. Ontology functional classification of genes with different transcript levels in the presence of 35 mM DMSO or nitrate.

Supplementary Figure 10. Validation of RNA-sequencing results.

Supplementary Tables:

Supplementary Table 1. Strains used in this study.

Supplementary Table 2. Plasmids and primers used in this study.

Supplementary Data:

S**upplementary Data 1.** Transcriptomic analysis of *S. algae* cultures supplemented with electron acceptors.

Supplementary Figure 1. Growth and biofilm formation patterns of *S. algae* strains in MB medium. Lateral and top views of strip well cultures (24 h, 30 ºC) before and after crystal violet staining of biofilms are shown.

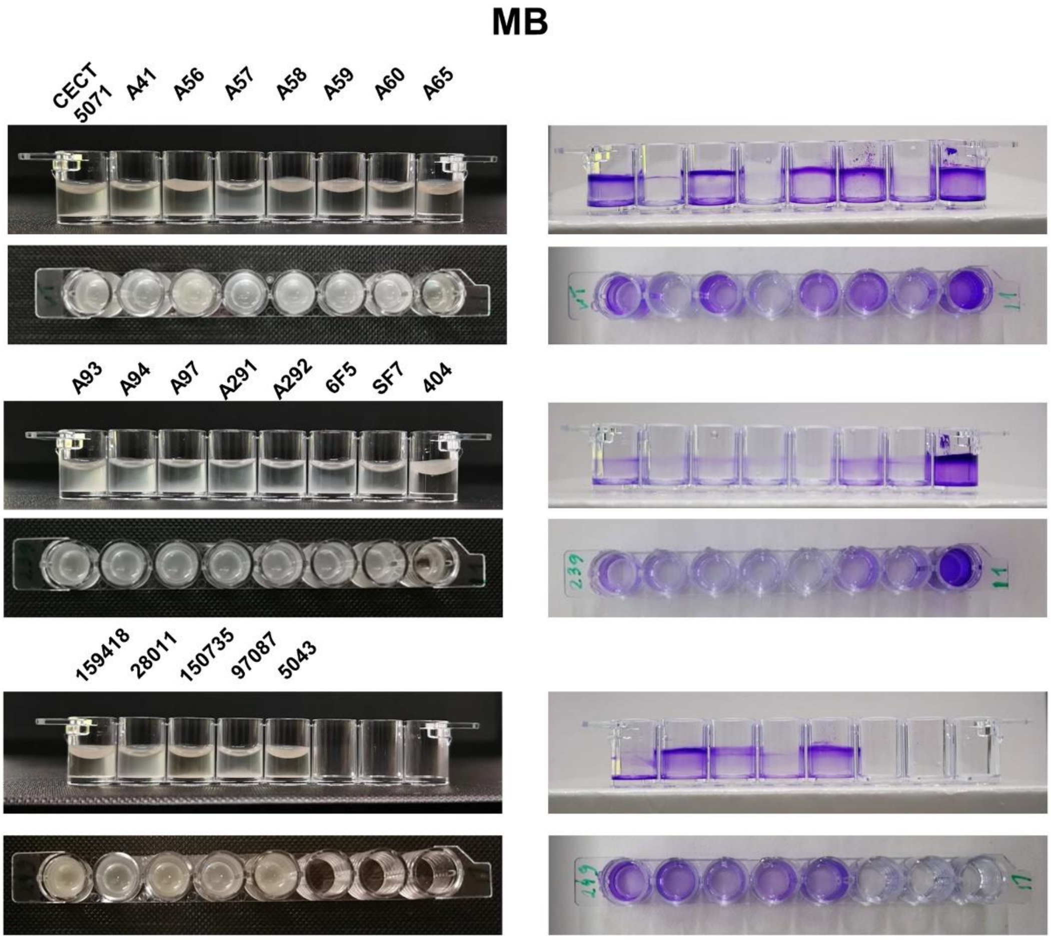

Supplementary Figure 2. Growth and biofilm formation patterns of *S. algae* strains in MB medium supplemented with 35 mM DMSO. Lateral and top views of strip well cultures (24 h, 30 ºC) before and after crystal violet staining of biofilms are shown.

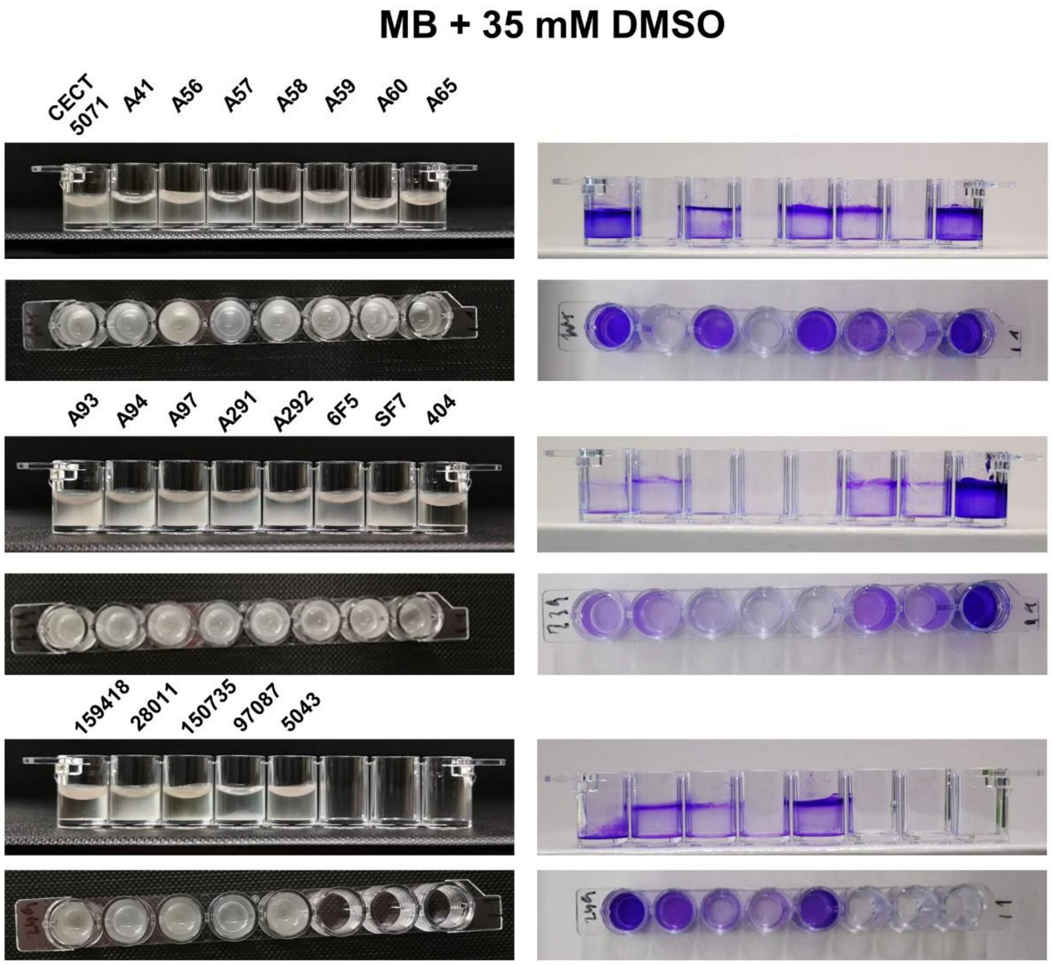

Supplementary Figure 3. Growth and biofilm formation patterns of *S. algae* strains in MB medium supplemented with 35 mM nitrate. Lateral and top views of strip well cultures (24 h, 30 ºC) before and after crystal violet staining of biofilms are shown.

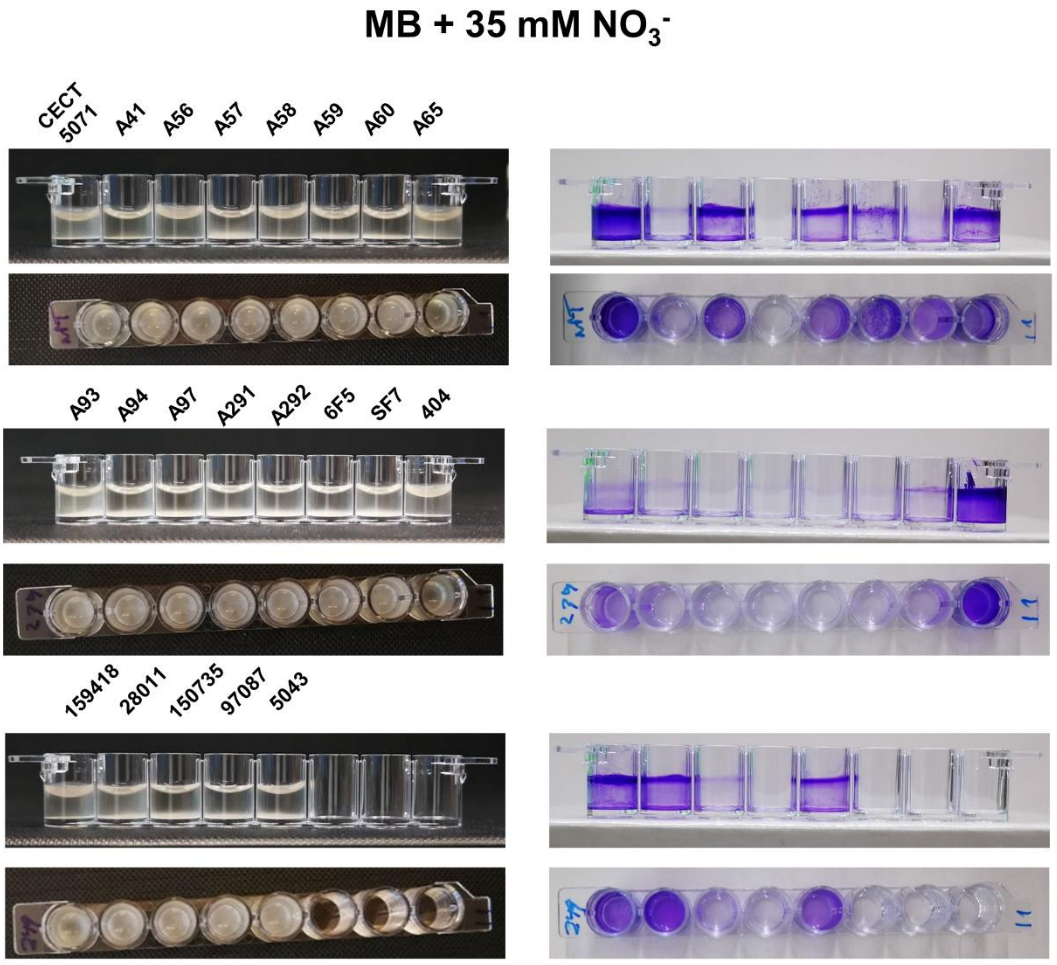

Supplementary Figure 4. Effect of pH changes on *S. algae* CECT 5071 biofilm formation. a. pH values (average ± SD, n = 3) of *S. algae* CECT 5071 cultures (24 h, 30 °C) in the absence or presence of 35 mM DMSO or nitrate in either MB or MB buffered by addition of 30 mM HEPES (pH = 7.5). **b.** Biofilm formation of *S. algae* CECT 5071 in the absence or presence of 35 mM DMSO or nitrate in buffered MB medium.

a

Condition	Not buffered	HEPES 30 mM
**MB (uninoculated)**	7.48±0.03	7.49±0.01
**MB + DMSO (uninoculated)**	7.50±0.01	7.47±0.01
**MB + nitrate (uninoculated)**	7.50±0.04	7.48±0.05
**CECT 5071 in MB**	6.73±0.08	7.28±0.01
**CECT 5071 in MB + 35 mM DMSO**	6.59±0.02	7.12±0.01
**CECT 5071 in MB + 35 mM nitrate**	6.35±0.12	6.88±0.01

b

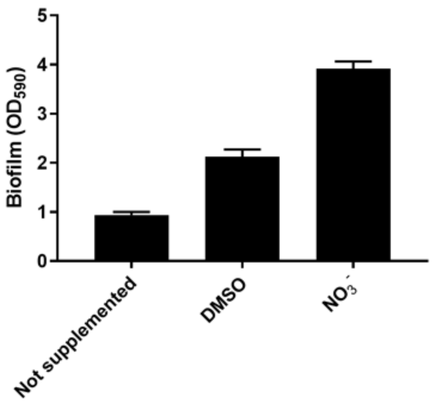

Supplementary Figure 5. Growth and biofilm formation patterns of *S. algae* WT and reductase mutants. a. Lateral and top views of strip well cultures (24 h, 30 ºC) of the WT strain and Δ*dmsB* mutant in the absence or presence of 35 mM DMSO before and after crystal violet staining of biofilms. **b.** Lateral and top views of strip well cultures (24 h, 30 ºC) of the WT strain and nitrate reductase mutants α^-, β^- and $\alpha\beta^-$ in the absence or presence of 35 mM sodium nitrate before and after crystal violet staining of biofilms. An additional top view of CV-stained wells held against a clear background shows more clearly differences in total biofilm biomass on the bottom of the wells.

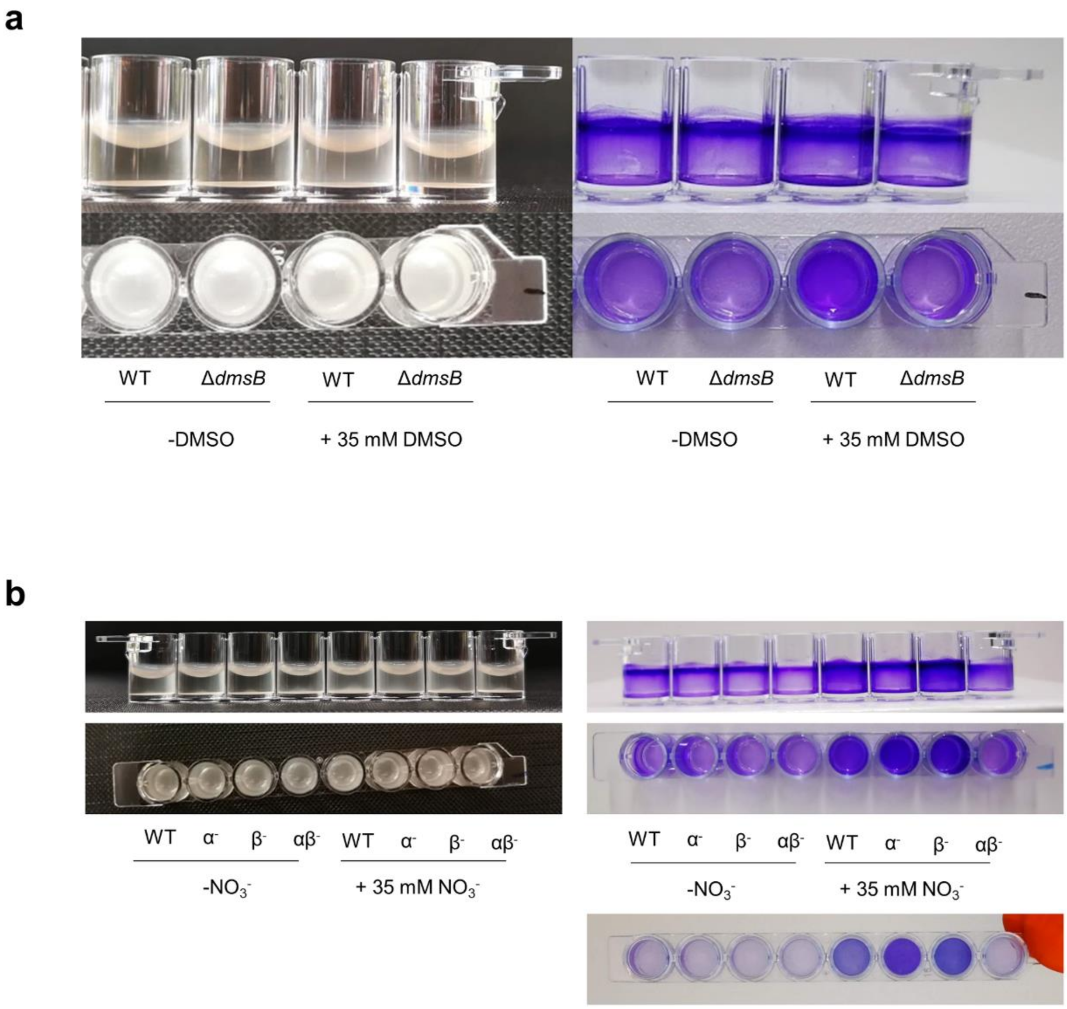

Supplementary Figure 6. Dose-dependent biofilm formation upon alternative electron acceptor supplementation in *S. algae* CECT 5071. a. Biofilm formation of *S. algae* CECT 5071 WT and Δ*dmsB* mutant in the absence or in the presence of two-fold serial dilutions of DMSO (0.55-70 mM). Data represent the average and SD of three biological replicates with three technical replicates per condition. **b.** Biofilm formation by *S. algae* CECT 5071 WT and nitrate reductase mutants α^-, β^- and $\alpha\beta^-$ in the absence or presence of two-fold serial dilutions of sodium nitrate (0.55-70 mM). Data represent the average and SD of three biological replicates with three technical replicates per condition.

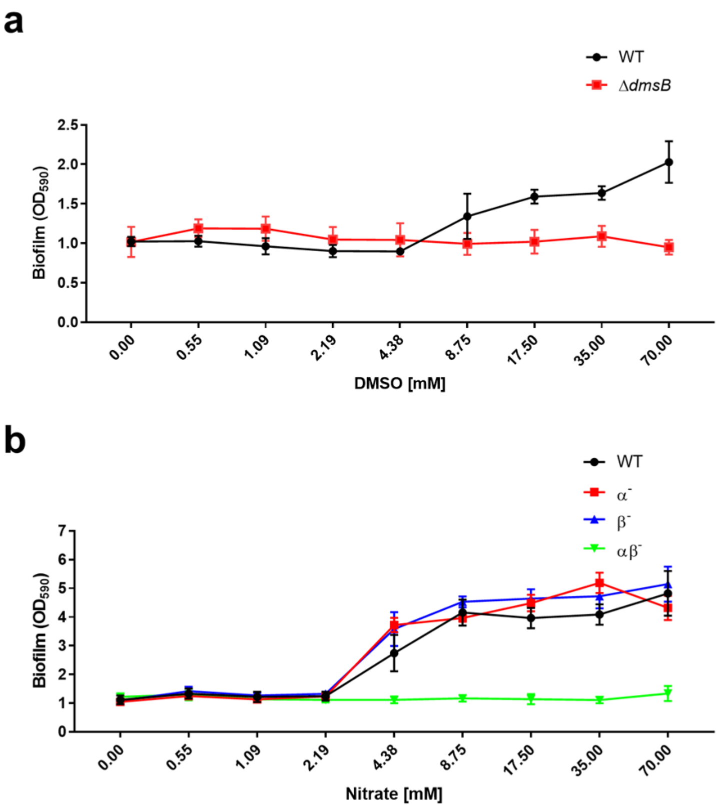

Supplementary Figure 7. Dimethylsulfide (DMS) detection in *S. algae* CECT 5071 static cultures supplemented with 35 mM DMSO. Chromatograms obtained by GC-MS for the WT strain harboring the empty pSRK-Km plasmid **(a)**, the Δ*dmsB* mutant harboring the empty pSRK-Km plasmid **(b)** and the Δ*dmsB* mutant complemented with the *dmsEFABGH* operon **(c)**.

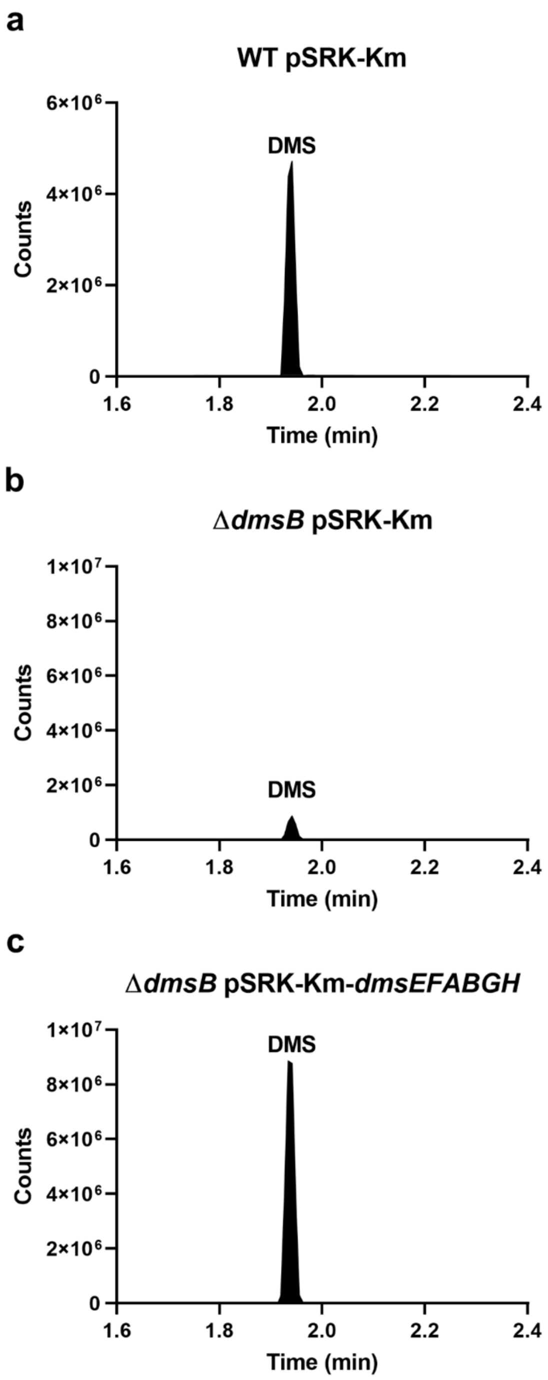

Supplementary Figure 8. Effect of plasmid pSRK-Km on *S. algae* CECT 5071 static biofilm formation. The figure shows CV staining of the biofilm formed by WT *S. algae* CECT 5071 and derivatives $\alpha\beta^-$, WT pSRK-Km and $\alpha\beta^-$ pSRK-Km grown in MB supplemented with 35 mM sodium nitrate. Note that the presence of plasmid pSRK-Km results in increased biofilm formation, particularly at the air-liquid interface.

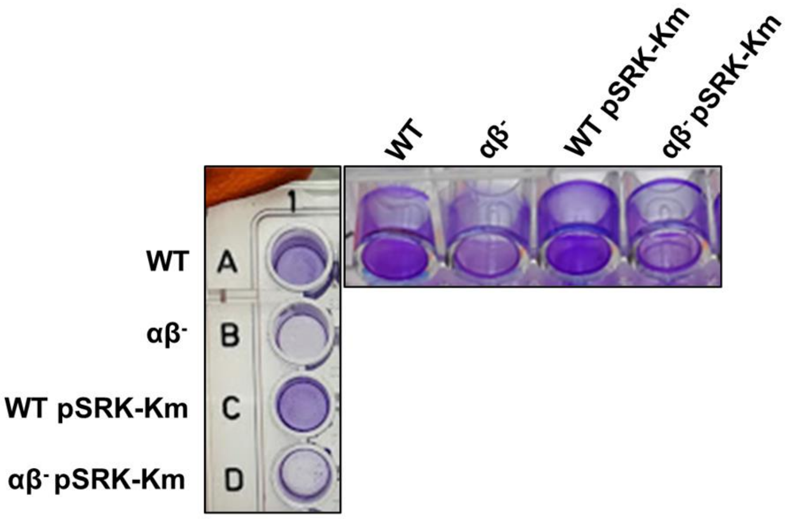

Supplementary Figure 9. Ontology functional classification of genes with different transcript levels in the presence of 35 mM DMSO or nitrate. Genes of the categories "biological processes" and "molecular functions" are shown for DMSO **(a-b)** and nitrate-supplemented cultures **(c-d)**.

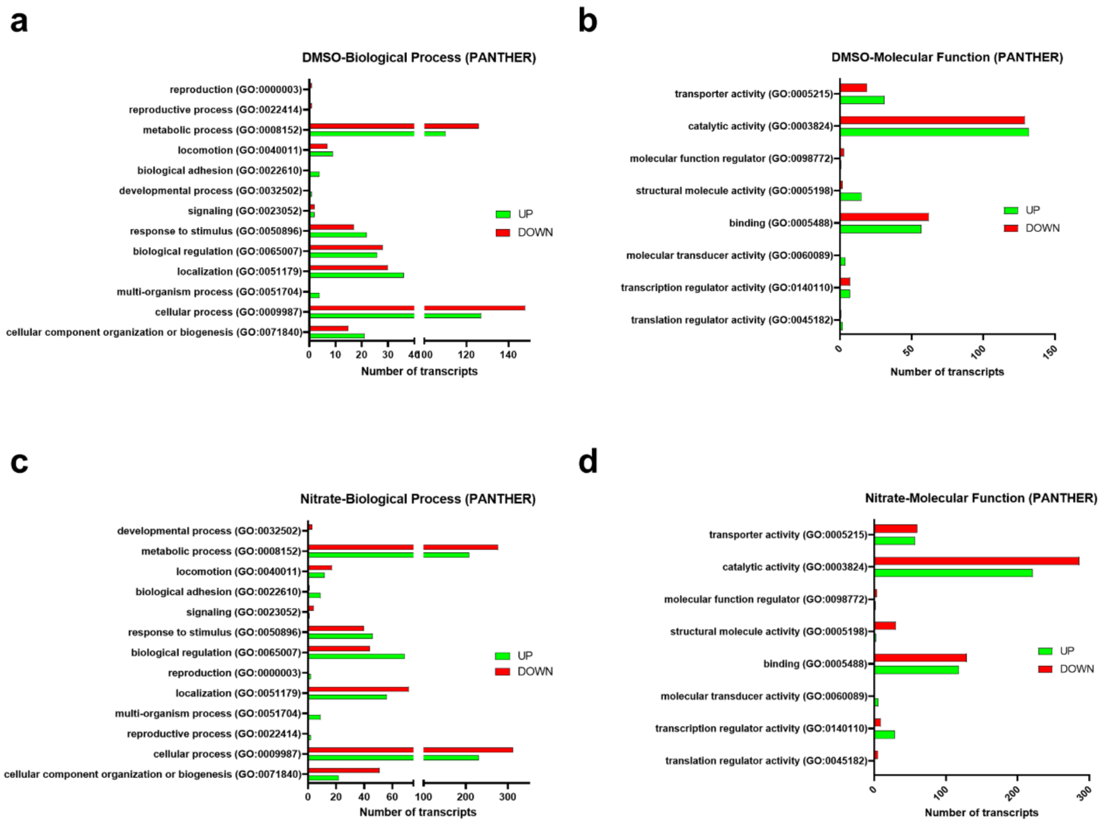

Supplementary Figure 10. Validation of RNA-sequencing results. Transcript levels of genes *dmsB* (E1N14_09855); *napA*-α (E1N14_17675); *napA*-β (E1N14_03175); *WT_00826* (E1N14_04075); *WT_00831* (E1N14_04100); and *WT_00655* (E1N14_03230) for **(a)** DMSO-supplemented cultures and **(b)** nitrate-supplemented cultures as determined by qRT-PCR or RNA-seq.

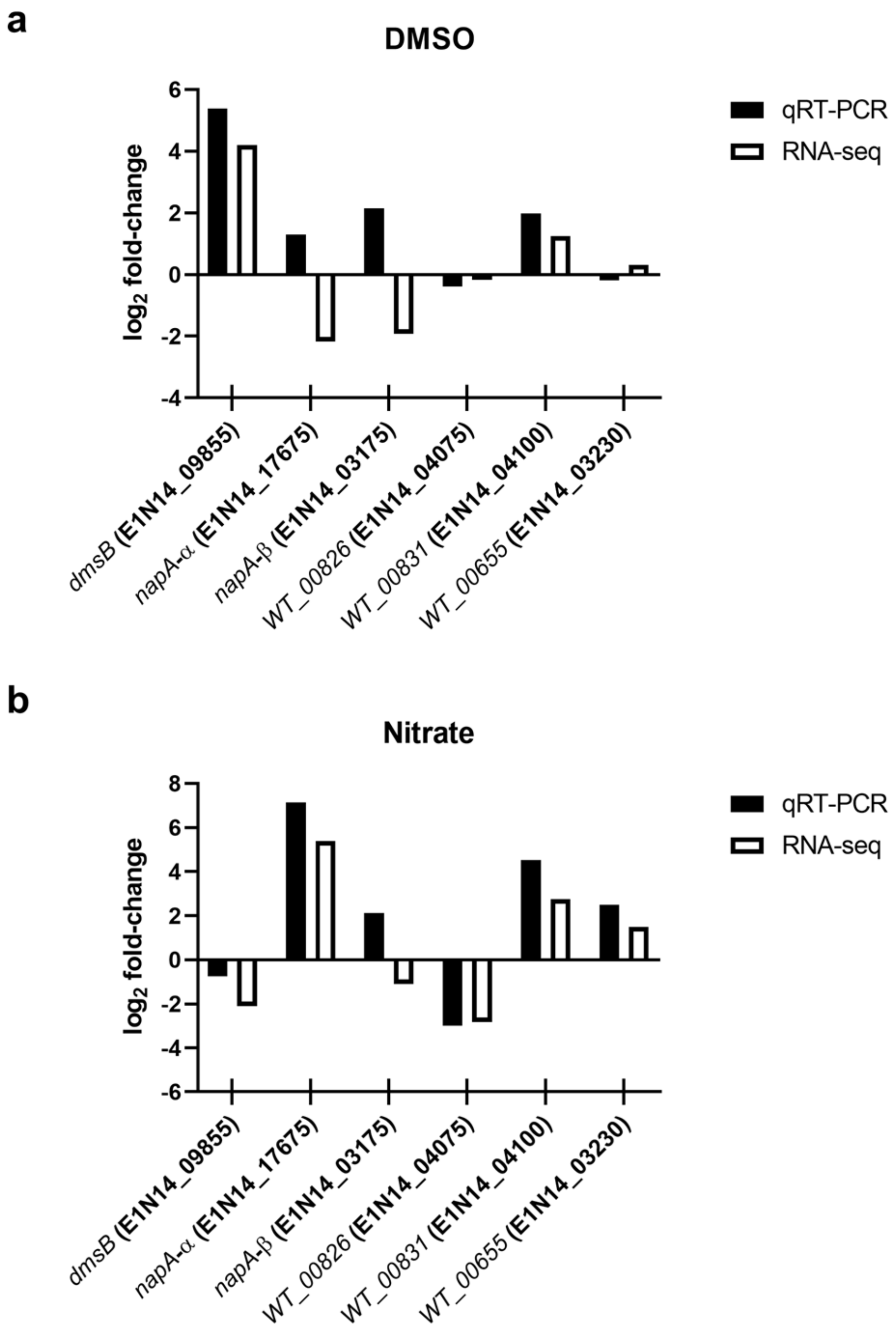

Supplementary Table 1. Strains used in this study.

**Strain***	**Description/source of isolation**	**Source or reference**
Escherichia coli **strains**		
Escherichia coli TOP10	F^- *mcrA* Δ(*mrr-hsdRMS-mcrBC*) φ80*lacZ*ΔM15 Δ*lacX*74 *rec*A1 *ara*D139 Δ(*ara-leu*)7697 *galU galK* λ^- *rpsL*(Str^R) *end*A1 *nupG*	Invitrogen
Escherichia coli NEB-5α	*fhuA2 (argF-lacZ)U169 phoA glnV44 80 (lacZ)*M15 *gyrA96 recA1 relA1 endA1 thi-1 hsdR17*	New England BioLabs
Escherichia coli DH5α λpir	*end*A1 *hsd*R17 *gln*V44 (= supE44) *thi*-1 *rec*A1 *gyr*A96 *rel*A1 φ80d*lac*Δ(*lac*Z)M15 Δ(*lacZYA-argF*)U169 *zdg*-232::Tn10 *uidA*::*pir+*	[1]
Escherichia coli MFDpir	MG1655 RP4-2-Tc::[ΔMu1::*aac*(3)IV-Δ*aphA*-Δ*nic35*-ΔMu2::zeo] Δ*dapA*::(erm-pir) Δ*recA*	[2]
Shewanella **strains**		
Shewanella algae CECT 5071 (ATCC 51192 = JCM 21037 = NBRC 103173)	Type strain, isolated from the surface of the red alga *Jania* sp. (Japan, 1990).	Spanish Type Culture Collection (CECT)
Shewanella algae A41 (NTCT10738 = LMG2265 =NCIMB11157)	Stool of a patient with acute enteritis (Japan, 1963)	[3]
Shewanella algae A56 (4.80-A)	Fish (Senegal, 1980)	[3]
Shewanella algae A57 (10.80-A)	Human wound (France, 1980)	[3]
Shewanella algae A58 (13.80-A)	Flamingo (France, 1980)	[3]
Shewanella algae A59 (14.80-A)	Flamingo (France, 1980)	[3]
Shewanella algae A60 (15.80-A)	Flamingo (France, 1980)	[3]
Shewanella algae A65 (2.82-B)	Poultry (France, 1982)	[3]
Shewanella algae A93 (43940)	Human blood (Denmark, 1994)	[3]
Shewanella algae A94 (48055)	Human blood (Denmark, 1994)	[3]
Shewanella algae A97 (AB2692)	Human wound (Denmark, 1992)	[3]
Shewanella algae A291	Cod (Denmark, 1995)	[3]

Shewanella algae A292	Plaice (Denmark, 1995)	3
Shewanella algae 6F5	Human bronchial aspirate (Gran Canaria, Spain, 2011)	3
Shewanella algae SF7	Human urethral catheter insertion exudate (Gran Canaria, Spain, 2010)	3
Shewanella algae 404	Cutaneous biopsy (Gran Canaria, Spain, 2008)	3
Shewanella algae 159418	Human ulcer (Gran Canaria, Spain, 2015)	3
Shewanella algae 28011	Human wound (Gran Canaria, Spain, 2014)	3
Shewanella algae 150735	Human wound (Gran Canaria, Spain, 2015)	3
Shewanella algae 97087	Human wound (Gran Canaria, Spain, 2015)	3
Shewanella algae 5043	Human wound (Gran Canaria, Spain, 2015)	3

*Synonymous strain codes are given in parentheses.

Supplementary Table 2. Plasmids and primers used in this study.

**Plasmid or primer**	**Characteristics or sequence**	**Source/reference**
**Plasmids**		
pUC18Not	Narrow-host range, high copy number cloning vector, P_{lac}, *lacZα*$^{+}$, Amp^{R}	[4]
pUC18-*dmsB*-UpDown	pUC18Not harboring a fusion of the chromosomal region 350 bp upstream and 351 bp downstream the *dmsB* gene, at HindIII/XbaI and XbaI/EcoRI sites, respectively	This study
pKNG101	suicide vector for allelic replacement, *sacB*^{R}, mobRK2, oriR6K, Sm^{R}	[5]
pKNG101-*dmsB*-UpDown	pKNG101 harboring a fusion of the chromosomal region 350 bp up- and 351 bp down-stream region of the *dmsB* gene, subcloned from pUC18-*dmsB* at the NotI site.	This study
pKNG101-NAP-α-UpDown	pKNG101 harboring a fusion of the chromosomal region 520 bp up- and 547 bp downstream the adjacent *napABC* genes from the Nap-α nitrate reductase, cloned at the SalI/BamHI and BamHI/SpeI sites, respectively.	This study
pKNG101-NAP-β-UpDown	pKNG101 harboring a fusion of the chromosomal region 500 bp up- and 500 bp downstream of *napA* of the Nap-β nitrate reductase, at BamHI/SalI and SalI/HindIII sites, respectively.	This study
pSRK-Km	Broad-host-range expression vector derived from pBBR1MCS-2, P_{lac}, *lacIq*, *lacZα*$^{+}$, Km^{R}	[6]
pSRK-Km-*dmsEFABGH*	pSRK-Km with the *dmsEFABGH* operon cloned into XbaI/XhoI sites, including the native putative Shine-Dalgarno sequence	This study
pSRK-Km-NAP-α	pSRK-Km with the *napEDABC* operon cloned into SalI/NheI sites, including the native putative Shine-Dalgarno sequence	This study
pSRK-Km-NAP-β	pSRK-Km with the *napDAGHB* operon cloned into XbaI/SalI sites, including the native putative Shine-Dalgarno sequence	This study
pSRK-Km-NAP-β-NAP-α	pSRK-Km with the *napDAGHB* and *napEDABC* operons cloned in tandem into XbaI/SalI and SalI/NheI sites, respectively, including the native putative Shine-Dalgarno sequences of both operons.	This study
pBBR1MCS-2	Broad-host range expression vector, P_{lac}, *lacZα*$^{+}$, Km^{R}	[7]
pBBR1MCS-2::*yhjH*	pBBR1MCS-2 with the *yhjH* gene from *Salmonella* Typhimurium cloned into HindIII/XbaI sites	This study

pBBR1MCS-2::*yhjH* E136A	pBBR1MCS-2 with a mutant *yhjH* gene from *Salmonella* Typhimurium cloned into HindIII/XbaI sites, resulting in the amino acid replacement E136A	[8]
**Primers for knockout generation***		
napA-beta-Up-F-BamHI	ATAGGATCCATATTGCAGGAGTCAAGCGG	This study
napA-beta-Up-R-SalI	CTAGTCGACGGTGTTTCCTCACTCTTTATTTCT	This study
napA-beta-Down-F-SalI	ATAGTCGACGGCAGCAGTAATGGGTGACA	This study
napA-beta-Down-R-HindIII	CTAAAGCTTTGATGGCCTCATCGATGAGC	This study
napABC-alpha-Up-F-SalI	ATAGTCGACACCCCTGCTGTCGGTTTTAC	This study
napABC-alpha-Up-R-BamHI	CTAGGATCCGGCGCCTTGTTCCAATCTA	This study
napABC-alpha-Down-F-BamHI	CTAGGATCC GCCCAAAGCTCTGGGCAG	This study
napABC-alpha-Down-R-SpeI	ATACTAGTCCTGCTAAGCTGACAATAGGTGT	This study
dmsB-Up-F-HindIII	ATAAAGCTTGTGCCCTGGCTGCGCGAAG	This study
dmsB-Up-R-XbaI	CTAGTCTAGATTTTTGATCCTCTATTATCTTTTCTTGA	This study
dmsB-DownF-XbaI	CTAGTCTAGACCCTCAAGAGAGCAGCCTT	This study
dmsB-DownR-EcoRI	AAGAATTCTAGCAGACTGATAAACCGAGCC	This study
**Primers for mapping in-frame gene deletion**		
dmsB-up-Up-F	ATGCATCCGGACATCAATTGG	This study
dmsB-down-Down-R	GAGCATGTTCTTCCGCCAGT	This study
napA-beta-up-Up-F	CATCACCATCAAGGGCACAC	This study
napA-beta-down-Down-R	ATCGCCGGTTCTTCCAAAAC	This study
napABC-alpha-Up-Up-F	TGCTGTATTTCATGTAAGCCTGA	This study
napABC-alpha-Down-Down-R	TGAATGGGGCTCACTGTTTA	This study
**Primers for genetic complementation**		

dms-XbaI-F	ATATCTAGATATAAATAAACGGGAGTACA	This study
dms-XhoI-R	TCACTCGAGTTAATATCTATTGTTGGTACTTATCA	This study
NAP-α-SalI-F	ATTGTCGACCATAGCGCCTAGGGAGGAGC	This study
NAP-α-NheI-R	TATGCTAGCTCAGAAGCCTTCCACTCCCT	This study
NAP-β-XbaI-F	TAATCTAGATACCTGTCGAGGTGAGCCTC	This study
NAP-β-SalI-R	ATAGTCGAC TCAGTTGGCAGCGCCAGT	This study
**Primers for the generation of point mutations**		
napA-α-PMs-F	TACTGGCTcCTCTGTGATGGTAGCCACCCGC	This study
napA-α-PMs-R	CCGgAAAAGCGGgACGGCGCCTTGTTCCAATC	This study
**Primers for *yhjH* cloning**		
yhjH-F-HindIII	ATAAAGCTTATGATAAAGCAGGTTATCCAGCA	This study
yhjH-R-XbaI	CTATCTAGATTACAGGGTCAGAATCACCTCT	This study
**Primers for qRT-PCR**		
qSA-*dmsB*-F	AGGTGCCATGCATAAACAGC	This study
qSA-*dmsB*-R	AACCGTCGCATTTGGTCATG	This study
qSA-*napA*- β -F	ACAAGCACACCAAGTTTGCC	This study
qSA-*napA*- β -R	ATCGCTGAACTTGCCGTTAC	This study
qSA-*napA*- α-F	ATTGTCTTCACGCCGCAAAC	This study
qSA-*napA*- α-R	TGCGGAAGTTCACATGCTTG	This study
qSA-*WT_00826*/ E1N14-4075-F	ACCAAAATCTGTGGCAAGGG	This study
qSA-*WT_00826*/ E1N14-4075-R	TTTTTCCAGCCGCTTGAAGC	This study
qSA-*WT_0831*/ E1N14-4100-F	AAAGTGTTGGCGGTGAACTC	This study
qSA-*WT_0831*/ E1N14-4100-R	ATTGGCTGCTGAACATGCTG	This study

qSA-*WT_0655*/ E1N14-3230-F	TTGTTGCTCTACCGTTTGCC	This study
qSA-*WT_0655*/ E1N14-3230-R	AAATTCCCAGCAGCACGATG	This study
qSA-*rpoD*-F	ACCATCAACAAGCTCAACCG	This study
qSA-*rpoD*-R	GGCGATTTTCAGCACTTTGC	This study

Supplementary Data 1. Transcriptomic analysis of *S. algae* cultures supplemented with electron acceptors. Differential gene expression of *S. algae* CECT 5071 cultures supplemented with 35 mM nitrate (columns I-P) or DMSO (columns R-Y) was calculated with respect to non-supplemented controls. Cluster of orthologous groups, PANTHER hits and KEGG orthology assigned for each gene are indicated, as well as shared differentially expressed genes based on a P-adj ≤ 0.05.

Supplementary references

1. Kolter, R., Inuzuka, M. & Helinski, D. R. Trans-complementation-dependent replication of a low molecular weight origin fragment from plasmid R6K. *Cell* **15**, 1199–1208 (1978).

2. Ferrières, L. *et al.* Silent mischief: Bacteriophage Mu insertions contaminate products of *Escherichia coli* random mutagenesis performed using suicidal transposon delivery plasmids mobilized by broad-host-range RP4 conjugative machinery. *J. Bacteriol.* **192**, 6418–6427 (2010).

3. Martín-Rodríguez, A. J., Suárez-Mesa, A., Artiles-Campelo, F., Römling, U. & Hernández, M. Multilocus sequence typing of *Shewanella algae* isolates identifies disease-causing Shewanella chilikensis strain 6I4. *FEMS Microbiol. Ecol.* **95**, fiy210 (2019).

4. Yanisch-Perron, C., Vieira, J. & Messing, J. Improved M13 phage cloning vectors and host strains: nucleotide sequences of the M13mp18 and pUC19 vectors. *Gene* **33**, 103–19 (1985).

5. Kaniga, K., Delor, I. & Cornelis, G. R. A wide-host-range suicide vector for improving reverse genetics in gram-negative bacteria: inactivation of the *blaA* gene of *Yersinia enterocolitica*. *Gene* **109**, 137–41 (1991).

6. Khan, S. R., Gaines, J., Roop, R. M., Farrand, S. K. & Farrand, S. K. Broad-host-range expression vectors with tightly regulated promoters and their use to examine the influence of TraR and TraM expression on Ti plasmid quorum sensing. *Appl. Environ. Microbiol.* **74**, 5053–62 (2008).

7. Kovach, M. E. *et al.* Four new derivatives of the broad host range cloning vector PBBR1MCS, carrying different antibiotic resistance cassettes. *Gene* **166**, 175–176 (1995).

8. Simm, R., Morr, M., Kader, A., Nimtz, M. & Römling, U. GGDEF and EAL domains inversely regulate cyclic di-GMP levels and transition from sessility to motility. *Mol. Microbiol.* **53**, 1123–34 (2004).
